# Contrasting shifts in potential climatic suitability of two *Thelazia callipaeda* vectors (*Phortica okadai* and *Phortica variegata*) across China and Europe under climate change

**DOI:** 10.1186/s13071-026-07417-x

**Published:** 2026-04-24

**Authors:** Yang Luo, Da Sun, Yajing Xu, Senqi Jia, Zhenfu Chen, Rong Yan, Juan Zhou, Bo Luo, Donghua Long, Rengze Yue, Domenico Otranto, Hui Liu, Lingjun Wang

**Affiliations:** 1 Department of Parasitology, Zunyi Medical University, Zunyi, China; 2 School of Laboratory Medicine, Zunyi Medical University, Zunyi, China; 3 Department of Veterinary Medicine, University of Bari, Valenzano, Italy; 4 Department of Veterinary Clinical Sciences, City University of Hong Kong, Hong Kong, China

**Keywords:** *Thelazia callipaeda*, *Phortica okadai*, *Phortica variegata*, MaxEnt, Species distribution modeling, Vector control

## Abstract

**Background:**

*Phortica okadai* and *Phortica variegata* are the primary vectors of the zoonotic eyeworm *Thelazia callipaeda*, which infects humans and various mammals. Climate change and intensified human activities have altered the potential suitable habitats of these vectors, posing a risk of expanded *T. callipaeda* transmission. This study aims to predict the current potential suitable habitats and future distribution patterns of the two species, providing a scientific basis for vector-borne disease prevention and control.

**Methods:**

Species occurrence records were compiled from the Global Biodiversity Information Facility (GBIF; https://www.gbif.org/) and systematic literature reviews. The MaxEnt model was utilized to identify key environmental determinants influencing vector distribution. Climate data from WorldClim, future climate scenarios (SSP1-2.6, SSP2-4.5, SSP5-8.5), elevation data, and Human Footprint Index (HFP) were integrated to predict potential suitable habitats and future distributions (2041–2060) across China and Europe.

**Results:**

The key environmental drivers for *P. okadai* are warmest quarter precipitation, HFP, and temperature seasonality, and for *P. variegata* they are HFP, coldest quarter precipitation, and temperature annual range. Currently, the suitable habitats of *P. okadai* are concentrated in central, eastern, and northeastern coastal China, with only sporadic low-suitability patches recorded in Europe. *P. variegata* exhibits a wide distribution across the UK, France, Belgium, and Italy, with nearly the entire Mediterranean coastal belt and its associated offshore islands falling within its suitable range. Under future climate scenarios, the suitable area of *P. okadai* is projected to expand significantly in Central/Western Europe (Italy, Austria, Switzerland, and western Russia). In contrast, the suitable habitats of *P. variegata* will shift significantly: The central–southern–eastern European transitional belt will lose almost all suitable habitat across scenarios, while the Mediterranean littoral and its offshore islands remain climatically suitable.

**Conclusions:**

The suitable area for *P. okadai* is projected to increase significantly, whereas that for *P. variegata* is expected to decline. Temperature and precipitation emerge as primary drivers of these contrasting distribution shifts. These findings underscore the need for enhanced vector surveillance and control strategies for *T. callipaeda*, particularly regarding the expanding *P. okadai* populations in Europe.

**Graphical Abstract:**

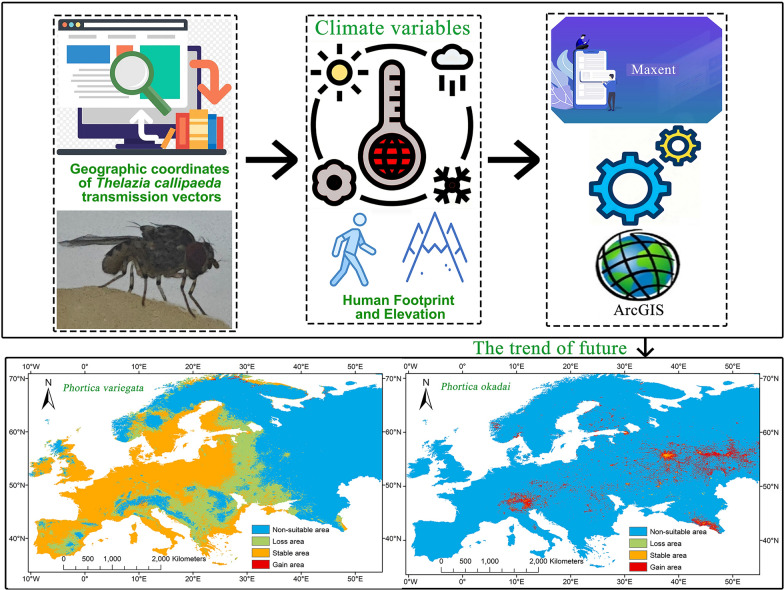

**Supplementary Information:**

The online version contains supplementary material available at 10.1186/s13071-026-07417-x.

## Background

*Thelazia callipaeda* (Spirurida: Thelaziidae) is the causative agent of thelaziasis, a parasitic infection of the ocular conjunctiva [1]. This nematode infects humans, companion animals (e.g., dogs and cats), and wildlife (e.g., foxes and bears), primarily inhabiting the nictitating membrane, conjunctival sac, and vitreous body. Clinical manifestations include conjunctival inflammation, retinal damage, vision loss, vitreous opacities, hypopyon, and endophthalmitis [2]. Current clinical management in humans primarily relies on surgical intervention, though complete removal of nematodes remains challenging [3]. Since the first human case was reported in China in 1917, *T. callipaeda* has been documented across East Asia and Europe, with recent reports from South Korea, Japan, India, Italy, Germany, and Portugal, suggesting potential for further range expansion under global warming [4–6]. Transmission is mediated by vectors, with *P. okadai* serving as the primary vector in Asia and *P. variegata* in Europe. These vectors acquire L1 larvae while feeding on ocular secretions of mammalian hosts and subsequently transmit them through their mouthparts [7, 8].

*P. okadai* was recently detected in Italy for the first time [9], whereas no *P. variegata* records exist for China. Given the profound impacts of climate change on existing habitats and the demonstrated adaptability of these species in newly invaded regions, urgent investigation of their potential suitable habitats and future distributions is warranted to inform proactive management strategies.

International trade has accelerated the introduction and spread of insect pests beyond their native geographic boundaries [10]. While trade facilitates global dissemination, establishment success in new environments depends critically on climatic suitability. Indeed, climate change creates novel dispersal pathways, likely accelerating the invasion and expansion of non-native insect species and potentially expanding the transmission range of *T. callipaeda* vectors. Species distribution modeling (SDM) has become an increasingly prevalent approach for investigating ecological niches of disease vectors and assessing altered outbreak risks under global climate change [11]. The close association of the nematode with its vector, *Phortica variegata* [8], and various hosts, allowed the preparation of ecological niche model for its distribution throughout Europe, using a desktop implementation of the Genetic Algorithm for Rule Set Prediction [12]. The model was based on ecological requirements of *P. variegata* across Europe, based on the observation that this drosophilid is mainly active at 20–25 °C and 50–75% relative humidity. The ecological niche model predicted vast areas suitable for *P. variegata* [12]. Consequently, based on the abovementioned scientific evidence and as a result of the increased awareness of practitioners and parasitologists, the number of reports of *T*. *callipaeda* infestation has increased in the last 20 years in areas where it was considered as nonendemic [1].

Among various niche modeling approaches, the maximum entropy (MaxEnt) model [13] has been widely applied in pest and disease control research owing to its robust performance even with small sample sizes [13–17]. MaxEnt integrates species occurrence data with environmental variables (e.g., climate, topography, and vegetation) to construct distribution models on the basis of the maximum entropy principle, minimizing the Kullback–Leibler divergence between actual species distribution and environmental background distribution [18, 19]. For instance, Yang et al. predicted expansion of *Bactrocera minax* distribution toward high-latitude and coastal regions under future climates [20], while Soliman et al. demonstrated habitat contraction for *Spogostylum ocyale* associated with greenhouse gas emissions [21].

This study represents the first application of MaxEnt modeling to predict potential suitable habitats and future distributions of *P. okadai* and *P. variegata* across China and Europe. Given the potential risk of *T. callipaeda* transmission to humans and animals via these vectors, defining their habitat suitability, future distribution dynamics, and ecological traits is crucial for developing scientifically sound control strategies. This research provides both a theoretical foundation for ecological management of these vectors and a robust scientific basis for future prevention and control of thelaziasis transmission.

## Methods

### Data collection and preparation

Species occurrence records for *P. okadai* and *P. variegata* were compiled from the Global Biodiversity Information Facility (GBIF; https://www.gbif.org/) and supplemented through a systematic literature review. To ensure the consistency of sampling protocols across data sources, we strictly filtered all raw records to retain only those derived from human observation (i.e., on-site field visual surveys and systematic sampling by professional investigators). For occurrence records of *P. okadai* (6 records) and *P. variegata* (12 records) lacking precise coordinates, locations were georeferenced to the centroid of the reported towns using Baidu Maps (http://map.baidu.com), as summarized in Supplementary Table S1. Two key considerations support the validity of this approach. First, given the relatively coarse spatial resolution of our environmental covariates (~4.6 km), our model inherently possesses a higher tolerance for positional errors compared with finer-scale (e.g., 100 m) scenarios [22]. Second, despite the maximum positional uncertainty of approximately 35 km associated with town centroids [23], this value is well within the widely accepted threshold of ten times the environmental resolution [24]. To minimize spatial bias, duplicate records within 2.5 arc-minute grid cells (~4.5 km^2^) were removed using ecological niche modeling tools (ENMTools) 1.0 in R, and ambiguous or incomplete entries were excluded following MaxEnt preprocessing protocols [25]. This resulted in 30 records for *P. okadai* and 108 for *P. variegata* for model construction (Supplementary Table S1) [4, 26–43].

### Selection of environmental variables

We selected 21 environmental variables that may influence the distribution of *P. okadai* and *P. variegata*. These include the 19 bioclimatic variables (WorldClim version 2.1, 1970–2000, 2.5 arc-minute resolution), future climate scenarios for 2041–2060 under three Shared Socioeconomic Pathways (SSP1-2.6, SSP2-4.5, and SSP5-8.5; WorldClim version 2.1, BCC-CSM2-MR model), elevation data (SRTM DEM version 3.0, NASA JPL, matching 2.5 arc-minute resolution), and Human Footprint Index (Global Human Footprint Index, CIESIN, 2023, 2.5 arc-minute resolution), which depicts the cumulative human pressure on the environment. Given the absence of consistent, high-resolution future projections of human footprint matching our study extent and SSP scenarios, the 2023 human footprint dataset was held constant in future habitat projections.

### MaxEnt model parameterization

The MaxEnt model (version 3.4.4) was implemented with a tenfold cross-validation framework as the core validation strategy to mitigate overfitting and assess the generalization ability of the model [13]. In each cross-validation round, the training subset of the current fold was further split randomly into 75% for model fitting and 25% for internal validation, to monitor model convergence and overfitting during the training process. The model was calibrated with the spatial domain for background point generation, covering the full global geographic range, while the formally delimited accessible area (the core study area for our distribution analysis and risk assessment) covered the full geographic scope of China and Europe, which fully covered all verified occurrence records of the two target species [44]. The model was run with a maximum of 10,000 background points randomly generated within the global spatial domain, default feature class combinations, and a regularization multiplier (RM) of 1. This parameter setting has been widely validated in previous studies to produce robust and non-overfitting predictive models for species with limited occurrence records [17, 45]. As described in the occurrence record preprocessing section, we removed duplicate records within each grid cell prior to model fitting; this operation mitigated spatial autocorrelation and sampling bias, and further ensured the independence of training and validation subsets, supporting the reliability of our random partitioning strategy.

### Model performance evaluation

Model predictive performance was assessed using three complementary metrics, with all values calculated on the basis of the held-out test fold of each tenfold cross-validation round; the final reported results are the mean ± standard deviation (SD) of the tenfold outputs. The three metrics are as follows: area under the receiver operating characteristic curve (AUC–ROC): a threshold-independent metric ranging from 0 (random discrimination) to 1 (perfect classification). Accuracy is categorized as: noninformative (0.0–0.6), marginal (0.6–0.7), moderate (0.7–0.8), substantial (0.8–0.9), and optimal (0.9–1.0) [46]. True skill statistic (TSS) is a threshold-dependent metric evaluating discriminatory power, with values spanning −1 (inverse agreement) to 1 (perfect agreement). Computed as TSS = sensitivity (true positive rate) + specificity (true negative rate) − 1, it mitigates prevalence bias effects. Performance thresholds are defined as: poor (0.0–0.4), fair (0.4–0.6), good (0.6–0.8), and excellent (0.8–1.0) [47]. Boyce index (BI) is a metric quantifying consistency between predicted habitat suitability and observed presence, ranging from −1 (inverse prediction) to 1 (perfect prediction). Calculated via Spearman rank correlation between predicted suitability bins and observed presence frequencies, values > 0.7 denote strong predictive performance [48]. Thresholds are categorized as: noninformative (≤ 0.0), marginal (0.0–0.2), moderate (0.2–0.5), substantial (0.5–0.7), and optimal (0.7–1.0). Unlike AUC–ROC, BI is robust to pseudo-absence selection and maintains sensitivity to spatial prediction quality. The predicted species distribution map represents habitat suitability as probability values ranging from 0 to 1, where values approaching 1 indicate a higher likelihood of species occurrence. Classification was implemented in ArcGIS via the natural breaks (Jenks) method [49]. Given the large number of suitable habitat zones in our study, we integrated threshold results from the natural breaks method across all scenarios to determine a consistent and appropriate classification scheme. We therefore adopted a four-category classification scheme: unsuitable (< 0.05), low (0.05–0.33), moderate (0.33–0.66), and high (≥ 0.66) [50].

### Statistical analysis of habitat suitability shifts

Future habitat shifts under SSP scenarios were quantified using ArcGIS reclassification tools. Zonal statistics were applied to compare current and projected distributions, identifying expansion or contraction trends in suitable areas.

## Results

### Model performance and key environmental variables

**Fig. 1:**
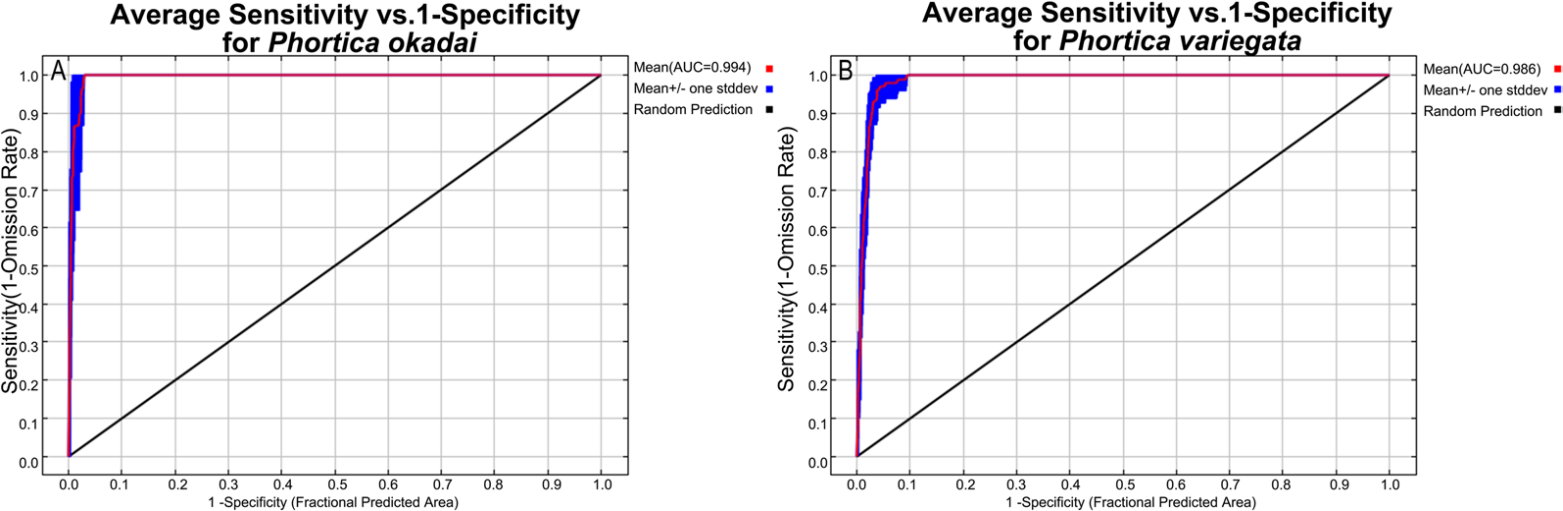
ROC curves and AUC values for MaxEnt model calibration. **A** *P. okadai*; **B** *P. variegata*

**Fig. 2:**
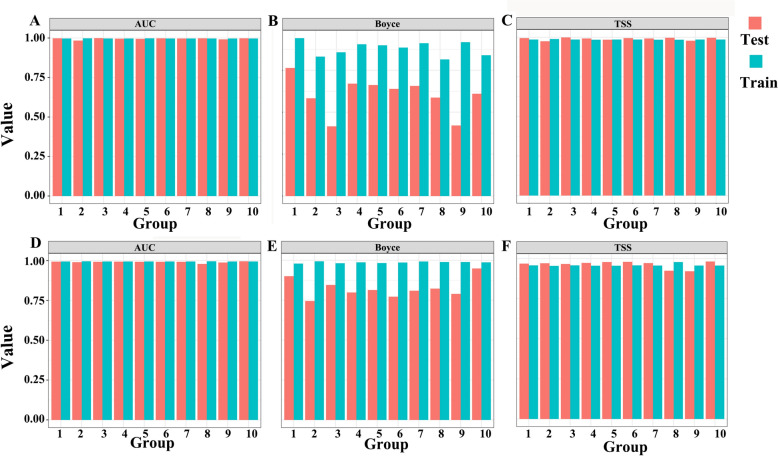
Performance evaluation of MaxEnt model for *P. okadai* and *P. variegata* via training and test set metrics. AUC (**A**, **D**), Boyce index (**B**, **E**), and TSS (**C**, **F**) values are shown for training (cyan) and test (red) datasets

The *P. okadai* model demonstrated exceptional performance: AUC–ROC reached 0.994 (optimal range) (Fig. 1), TSS was 0.988 ± 0.004 (excellent range), and BI was 0.741 ± 0.065 (optimal range) (Fig. 2). The *P. variegata* model also exhibited outstanding performance: AUC–ROC 0.986 (optimal range), TSS 0.990 ± 0.009 (excellent range), and BI 0.601 ± 0.109 (substantial range). The mean TSS values for both species exceeded 0.7, meeting high-precision criteria; BI values > 0.6 further validated strong correlation between predicted habitat suitability and observed distributions. Percent contribution analysis identified critical environmental determinants for each species’ distribution model. For *P. okadai*, warmest quarter precipitation (Bio 18) contributed most (32.6%), followed by HFP (30.9%) and temperature seasonality (Bio 4) (14.6%). Precipitation seasonality (Bio 15) contributed 4.7%, while mean temperature of driest quarter (Bio 9) and coldest quarter (Bio 11) contributed 3.9% and 3.4%, respectively. The top six variables (Bio 4, Bio 9, Bio 11, Bio 15, Bio 18, and HFP) cumulatively accounted for 90.1% of explained variance.

**Fig. 3:**
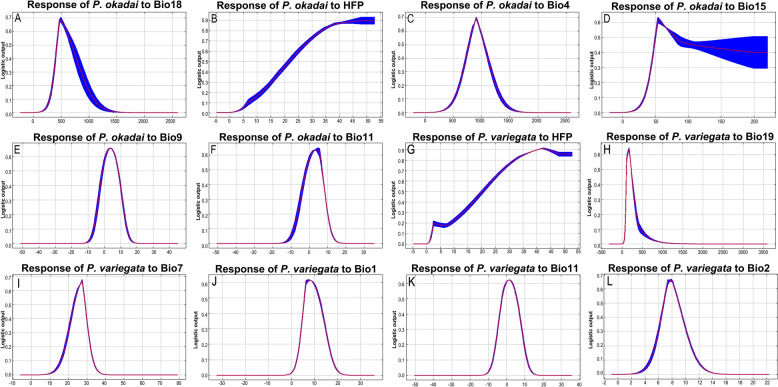
Response curves for key environmental variables in MaxEnt models. Blue shading indicates ±1 standard deviation; red line represents mean response. **A–F** *P. okadai* (**A** Bio 18; **B** HFP; **C** Bio 4; **D** Bio 15; **E** Bio 9; **F** Bio 11); **G–L** *P. variegata* (**G** HFP; **H** Bio 19; **I** Bio 7; **J** Bio 1; **K** Bio 11; **L** Bio 2)

For *P. variegata*, HFP was the most important predictor (28.6%), followed by precipitation of coldest quarter (Bio 19) (17.2%) and temperature annual range (Bio 7) (15.4%). Annual mean temperature (Bio 1) contributed 9.6%, mean temperature of coldest quarter (Bio 11) (4.6%), and mean diurnal temperature range (Bio 2) (3.2%). The top six variables (Bio 1, Bio 2, Bio 7, Bio 11, Bio 19, and HFP) collectively explained 78.6% of variance. Response curves (Fig. 3) illustrate optimal ranges for these key variables (Table 1).

**Table 1:** Key environmental variable influencing distribution of *P. okadai* and *P. variegata*

*P. okadai*/*P. variegata*	Environmental variable	Percent contribution (%)	Suitable ranges	Best survival point
1	Bio 18/HFP	32.60/28.60	425–692 mm/≥ 20 (dimensionless)	490 mm/42 (dimensionless)
2	HFP/Bio 19	30.90/17.20	≥ 22 (dimensionless)/109–227 mm	48 (dimensionless)/176 mm
3	Bio 4/Bio 7	14.60/15.40	771–1043 (dimensionless)/23–29 °C	900 (dimensionless)/27 °C
4	Bio 15/Bio 1	4.70/9.60	50–80 (dimensionless)/6–12 °C	54 (dimensionless)/8 °C
5	Bio 9/Bio 11	3.90/4.60	−1 to 8 °C/−3 to 6 °C	4 °C/1 °C
6	Bio 11/Bio 2	3.40/3.20	−2 to 7 °C/7 to 9 °C	4 °C/8 °C

Bio 18, precipitation of warmest quarter; HFP, human footprint; Bio 4, temperature seasonality; Bio 15, precipitation seasonality; Bio 9, mean temperature of driest quarter; Bio 11, mean temperature of coldest quarter; Bio 19, precipitation of coldest quarter; Bio 7, temperature annual range; Bio 1, annual mean temperature; Bio 2, mean diurnal temperature range

### Current potential suitable areas in China (1970–2020)

**Fig. 4:**
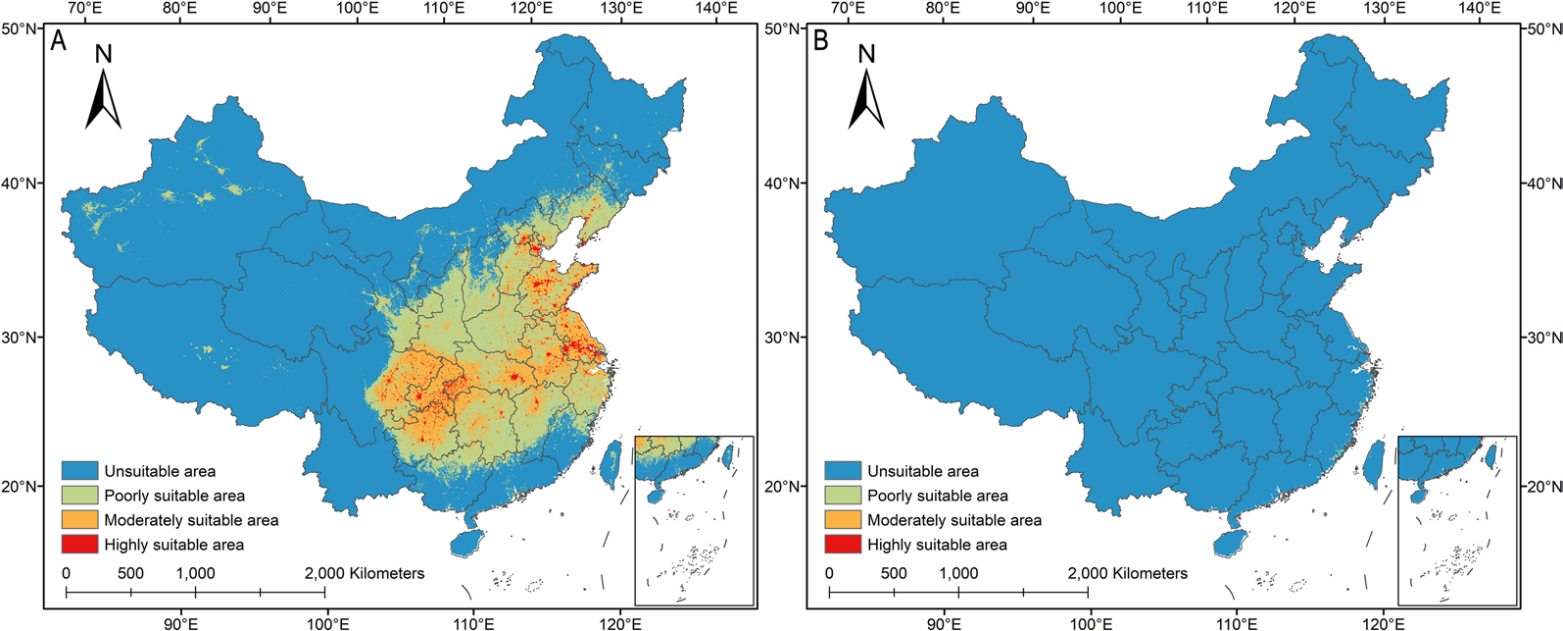
Current (1970–2020) suitable climatic distribution of *P. okadai* and *P. variegata* in China. **A** *P. okadai*; **B** *P. variegata*

MaxEnt projections for China (Fig. 4) indicated that potentially suitable habitats for *P. okadai* were concentrated in eastern and central regions, including the provinces of Guizhou, Hunan, Jiangxi, Zhejiang, eastern Sichuan, Chongqing, Hubei, Anhui, Jiangsu, Shaanxi, Henan, Shandong, Shanxi, Beijing, and Tianjin, with additional presence in Liaoning (Northeast China) and scattered patches in northwestern Xinjiang and central Tibet. By comparison, extensive low-suitability habitats of *P. variegata* occurred along the coastal zone linking Fujian, Zhejiang, and Taiwan, whereas only scattered, minimal low-suitability patches were projected in Guizhou, Chongqing, Jiangxi, Hunan, and Guangdong.

### Future suitable areas in China (2041–2060)

**Fig. 5:**
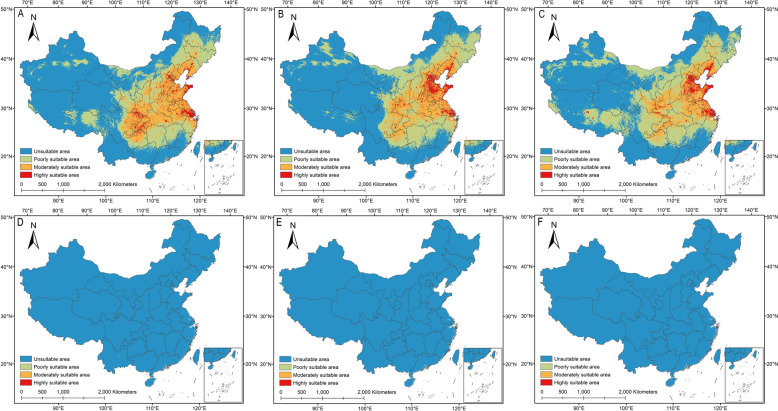
Future (2041–2060) suitable climatic distribution of *P. okadai* and *P. variegata* in China under three SSP scenarios. **A–C** *P. okadai* (**A** SSP1-2.6; **B** SSP2-4.5; **C** SSP5-8.5); **D–F** *P. variegata* (**D** SSP1-2.6; **E** SSP2-4.5; **F** SSP5-8.5)

**Fig. 6:**
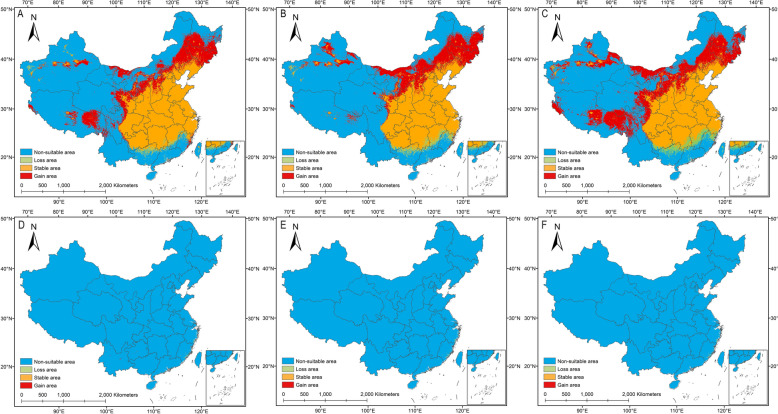
Distribution change trends in suitable habitats for *P. okadai* and *P. variegata* in China. **A–C** *P. okadai* (**A** SSP1-2.6; **B** SSP2-4.5; **C** SSP5-8.5); **D–F** *P. variegata* (**D** SSP1-2.6; **E** SSP2-4.5; **F** SSP5-8.5)

Future projections (Fig. 5) revealed that suitable areas for *P. okadai* will increase across all three climate scenarios (Table 2). Under SSP1-2.6, highly suitable habitats will expand significantly in eastern Sichuan, northern Guizhou, Chongqing, Shandong Peninsula, and southern Jiangsu/Shanghai. Under SSP2-4.5 and SSP5-8.5, substantial increases occurred in the Liaodong Peninsula, Beijing–Tianjin–Hebei region, and southern Jiangsu. In contrast, the overall suitable area for *P. variegata* in China is projected to contract; the habitat belt between Fujian and Taiwan virtually disappears, and under SSP2-4.5, the entire Chinese range of this species will be lost. Under SSP1-2.6, however, small, newly emerged low-suitability pockets arise in Guizhou and Yunnan. Distribution change maps (Fig. 6) demonstrated a clear northward expansion trend in suitable areas for *P. okadai*, with minor losses in southern regions.

**Table 2:** Current and projected changes (2041–2060) in suitable areas for *P. okadai* and *P. variegata* in China (km^2^)

Decade	Scenarios	Predicted area (10^3^ km^2^)			Comparison with current distribution (%)		
		High suitability	Moderate suitability	Low suitability	High suitability	Moderate suitability	Low suitability
Current	*P. okadai*	94.23	773.24	1584.21	N/A	N/A	N/A
	*P. variegata*	0.00	0.00	3.82	N/A	N/A	N/A
*P. okadai* 2050s	SSP1-2.6	254.00	1363.91	2195.90	+169.6	+76.3	+38.6
	SSP2-4.5	274.23	1211.99	2274.36	+191.1	+56.8	+43.6
	SSP5-8.5	255.12	1136.47	2898.26	+170.8	+47.0	+83.0
*P. variegate* 2050s	SSP1-2.6	0.00	0.00	1.95	N/A	N/A	−49.0
	SSP2-4.5	0.00	0.00	0.00	N/A	N/A	N/A
	SSP5-8.5	0.00	0.00	0.21	N/A	N/A	−94.5

N/A, not available

### Current potential suitable areas in Europe (1970–2020)

**Fig. 7:**
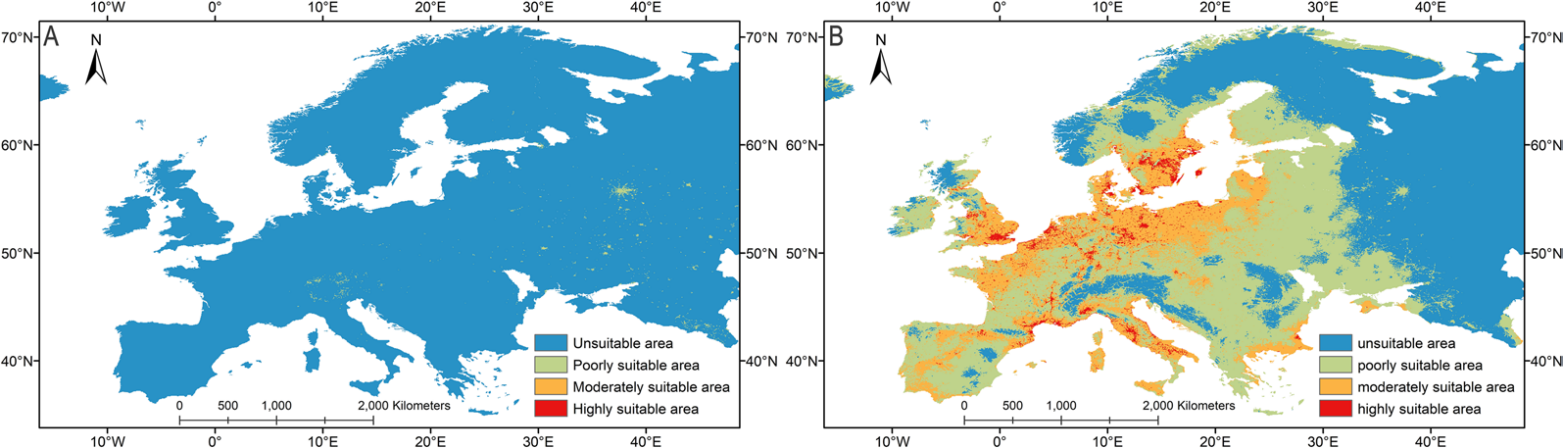
Current (1970–2020) suitable climatic distribution of *P. okadai* and *P. variegata* in Europe. **A** *P. okadai*; **B** *P. variegata*

European projections (Fig. 7) showed sporadic, low-suitability habitats for *P. okadai* in Switzerland, Italy, Austria, Ukraine, and western Russia, with restricted overall extent (Table 3). *P. variegata*, however, exhibited broad distribution across the UK, France, Belgium, the Netherlands, Italy, Sweden, and the European part of Turkey, with virtually the entire Mediterranean coastal belt and its offshore islands falling within its suitable range.

**Table 3:** Current and projected changes (2041–2060) in suitable areas for *P. okadai* and *P. variegata* in Europe (km^2^)

Decade	Scenarios	Predicted area (10^3^ km^2^)			Comparison with current distribution (%)		
		High suitability	Moderate suitability	Low suitability	High suitability	Moderate suitability	Low suitability
Current	*P. okadai*	0.00	0.00	40.96	N/A	N/A	N/A
	*P. variegata*	158.05	1319.08	3163.36	N/A	N/A	N/A
*P. okadai* 2050s	SSP1-2.6	0.04	4.68	250.08	N/A	N/A	+510.6
	SSP2-4.5	0.01	11.19	464.73	N/A	N/A	+1034.6
	SSP5-8.5	0.00	11.81	638.95	N/A	N/A	+ 1459.9
*P. variegata* 2050s	SSP1-2.6	34.59	457.08	2767.13	−78.1	−65.3	−12.5
	SSP2-4.5	39.88	623.75	2426.75	−74.8	−52.7	−23.3
	SSP5-8.5	26.23	396.21	2501.41	−83.4	−70.0	−20.9

N/A, not available

### Future suitable areas in Europe (2041–2060)

**Fig. 8:**
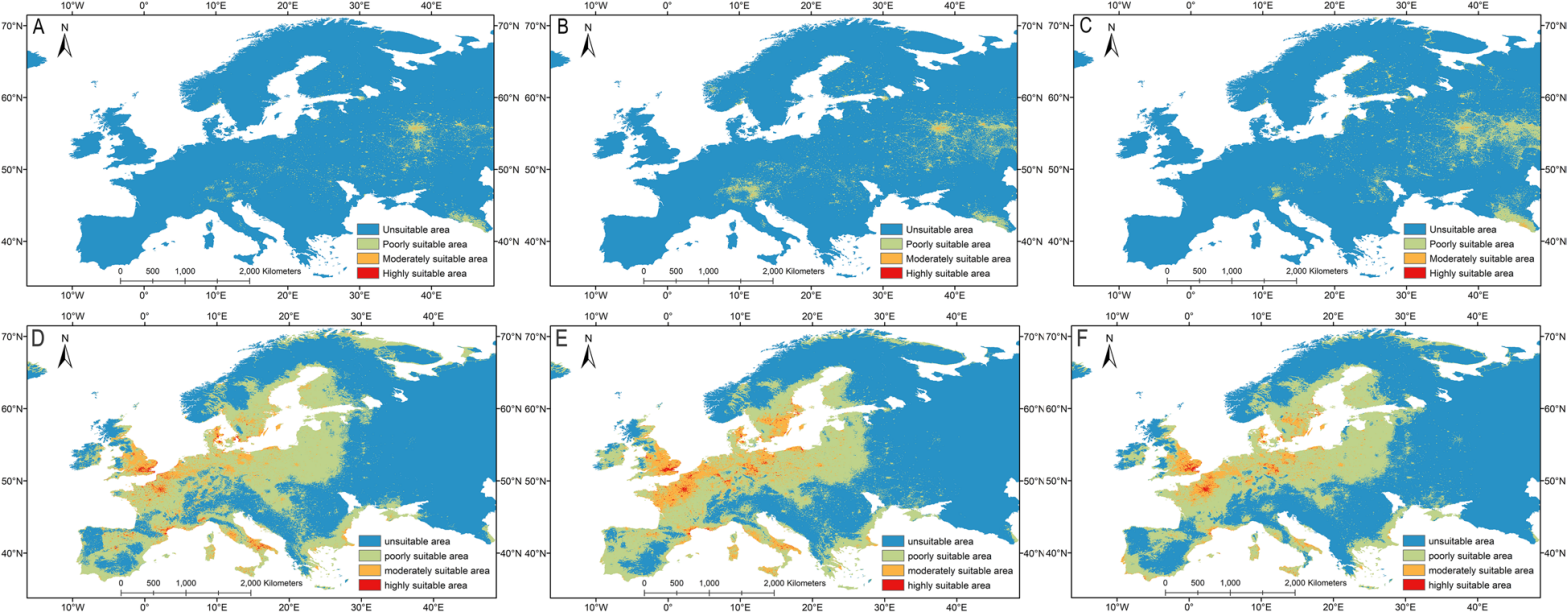
Future (2041–2060) suitable climatic distribution of *P. okadai* and *P. variegata* in Europe under three SSP scenarios. **A**–**C** *P. okadai* (**A** SSP1-2.6; **B** SSP2-4.5; **C** SSP5-8.5); **D**–**F** *P. variegata* (**D** SSP1-2.6; **E** SSP2-4.5; **F** SSP5-8.5)

**Fig. 9:**
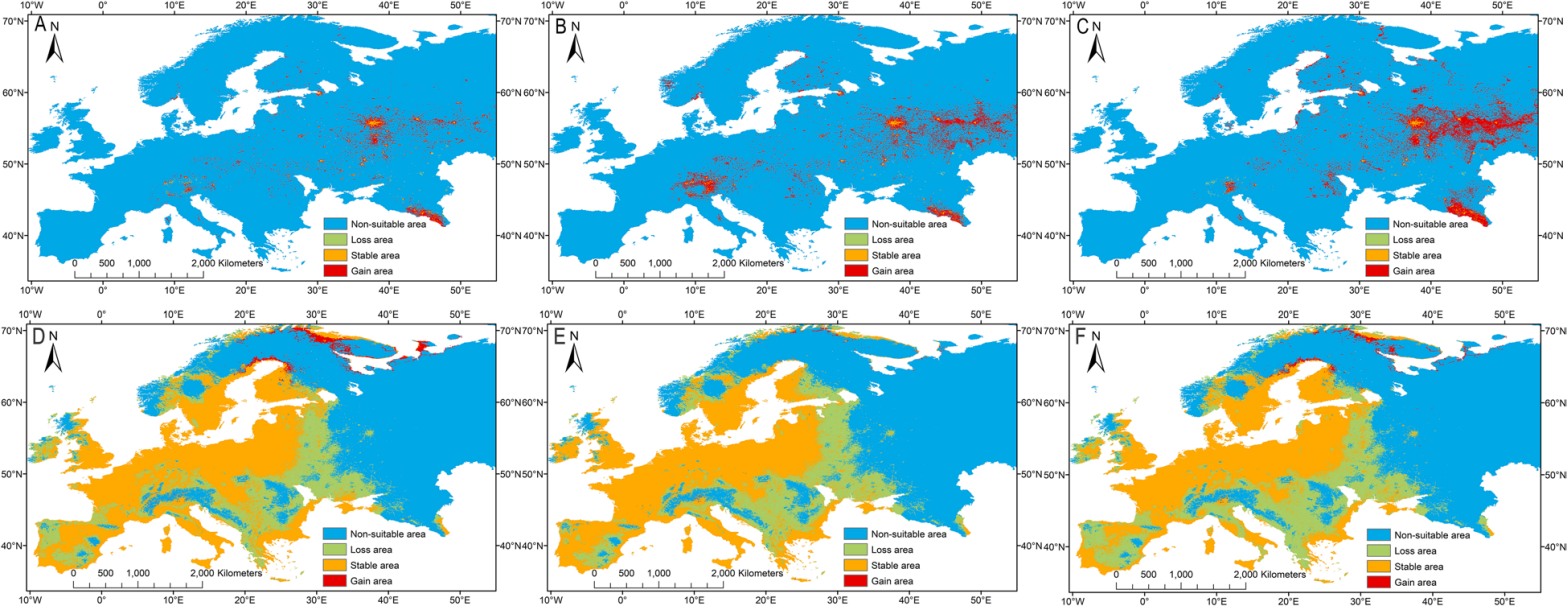
Distribution change trends for suitable habitats in Europe. **A**–**C** *P. okadai* (**A** SSP1-2.6; **B** SSP2-4.5; **C** SSP5-8.5); **D**–**F**: *P. variegata* (**D** SSP1-2.6; **E** SSP2-4.5; **F** SSP5-8.5)

Under future climate scenarios (Fig. 8), the potential climatically suitable area for *P. okadai* showed a substantial increase, with emergence of moderate-suitability zones. However, these areas represent climate potential only; actual establishment will depend on dispersal ability and biotic interactions. Conversely, the climatically suitable area for *P. variegata* contracted markedly. Across the three future scenarios, high-suitability habitat declined by up to 83.4% under SSP5-8.5, moderate-suitability habitat declined by at least 52.7% under SSP2-4.5, and low-suitability habitat declined minimally—by 12.5%—under SSP1-2.6 (Table 3). Distribution change maps (Fig. 9) revealed that *P. okadai* expansion concentrated in western Russia and northern Greater Caucasus regions, with sporadic increases across central Europe. Notably, under SSP2-4.5, significant expansion of suitable habitats is projected for border regions of Italy, Switzerland, and Austria, while SSP5-8.5 shows the most substantial overall increases. For *P. variegata*, the transitional belt linking Central, Southern, and Eastern Europe is projected to lose virtually all suitable habitat, whereas the Mediterranean littoral and its offshore islands remain climatically suitable across all scenarios.

## Discussion

This study represents the first integrated analysis of *P. okadai* and *P. variegata* distribution patterns using MaxEnt modeling with comprehensive environmental variables. Warmest quarter precipitation (Bio 18), human footprint (HFP), and temperature seasonality (Bio 4) are identified as primary determinants for *P. okadai*, whereas HFP, precipitation of coldest quarter (Bio 19), and temperature annual range (Bio 7) critically influence *P. variegata*. These divergent environmental associations may be related to the evolutionary adaptation of the two species to their native climatic regimes.

For *P. okadai*, habitat suitability peaks at 0.69 when warmest quarter precipitation (Bio 18) reaches 490 mm, with a sharp decline observed when this precipitation falls below 400 mm—indicating a strong association between the occurrence of *P. okadai* and summer rainfall, which may represent a key environmental constraint on the species’ distribution. Consistent with this climatic preference, *P. okadai* is more prevalent in hot, humid summer conditions and can complete the full life cycle under moderately cold winter temperatures (3–15 °C) [27], indicating adaptability to non-extreme seasonal cold. In addition, *P. okadai* exhibits specific adaptation to temperature seasonality (Bio 4; expressed as SD × 100), with a suitable range of 771–1043 and maximum habitat suitability at a Bio 4 value of 900 (equivalent to a 9 °C standard deviation in monthly mean temperatures). This trait reflects the tolerance of *P. okadai* for pronounced seasonal thermal fluctuations in native habitats of the species [51]. Currently, suitable habitats of *P. okadai* are concentrated in central, eastern, and northeastern China (Fig. 4A)—a region where summer precipitation constitutes 60–80% of annual rainfall [52]. This climatic characteristic matches the optimal Bio 18 range (425–692 mm), with habitat suitability peaking at 490 mm for *P. okadai*, indicating a strong relationship between climatic suitability and summer precipitation.

In contrast, *P. variegata*, native to the Mediterranean climate zone of Europe, relies primarily on coldest quarter precipitation (Bio 19, 17.2% contribution), aligning with the mild, wet winters and hot, dry summers of this region. Current widespread suitable habitats for *P. variegata* in central Europe (Fig. 7B) overlap with areas where winter precipitation (December–February) sustains overwintering stages (pupae or adults) [12, 53]. The optimal Bio 19 range for *P. variegata* (109–227 mm) matches the typical winter rainfall of the Mediterranean basin, while habitat suitability declines sharply below 109 mm—indicating that winter moisture is closely associated with habitat suitability for the population persistence of the species. This association aligns with the contrasting projected distribution shifts: *P. okadai* shows a positive response to enhanced summer precipitation in Central/Western Europe under climate change (Fig. 9A–C), corresponding to a 510.6–1459.9% expansion of its climatically suitable area (Table 3). Conversely, *P. variegata* exhibits a negative association with reduced winter precipitation across all climate scenarios (Fig. 9D–F),which may be associated with reduced suitability for overwintering conditions in the transitional belt linking Central, Southern, and Eastern Europe. It is critical to note that these patterns reflect statistical associations derived from the MaxEnt model rather than direct ecological causality; further field validation is required to confirm the biological mechanisms underlying these climate–distribution relationships.

The exceptionally high AUC (> 0.98) and TSS (> 0.98) values should be interpreted with caution, as they may partly reflect the large background extent and associated environmental contrasts rather than solely the model’s predictive performance. The background extent used for model training covers a broad geographic range (China and Europe), which increases environmental divergence between species occurrence points and background pixels, simplifying the discrimination task [44, 47]. In contrast, the moderate to high Boyce index (BI) provides a more ecologically meaningful assessment of model performance, as it is independent of background selection and quantifies the consistency between predicted habitat suitability and observed species occurrences [48]. Although the limited sample size in this study prevents a full characterization of the species’ entire climatic niche, the favorable BI performance, combined with the inherent advantages of MaxEnt for small sample sizes, supports the reasonable reliability of suitable habitat predictions for the two species.

HFP emerged as a significant shared predictor, consistent with the life history of these vectors. Both species frequent areas with intensive agricultural activities [37, 53], are commonly associated with orchards and oak woodlands [43], and exhibit zoophilic behavior by feeding on tear secretions of humans and animals [40, 54]. Global trade may facilitate their long-distance dispersal via fruit shipments [55, 56], consistent with the recent detection of *P. okadai* in Italy [9], and the predicted suitable habitats in this study align with this observed distribution record. Notably, the future distribution projections in this study only incorporated the current human footprint dataset, without accounting for potential future changes in human activity intensity and land use patterns under socioeconomic development scenarios. This represents an important source of uncertainty in these future projections, as changes in human activities may significantly alter the actual suitable habitats of the two species, independent of climate change effects [57]. Minor geographic discrepancies between the predicted and observed distributions (Supplementary Fig. S2) may reflect three key limitations of the model calibration process: limited occurrence records, unaccounted biotic interactions (e.g., competition, predation) [58, 59], and inherent uncertainty of climate variables. As demonstrated by Fick et al., historical climate data exhibit precision errors, particularly in regions with sparse meteorological stations [59], which may further influence the reliability of model outputs. In addition, the future climate projections in this study are based on a single global climate model (BCC-CSM2-MR). While this model is widely validated and applied in regional climate change studies in Asia and Europe, reliance on a single global climate model (GCM) limits the assessment of climatic uncertainty in this work: Different CMIP6 models can produce substantially different regional projections of temperature and precipitation, and the results presented here cannot capture the full spectrum of future climate change uncertainty [60]. The inherent extrapolation risk associated with these model projections is also noted: When transferring the calibrated model to future climatic conditions, a subset of predictions may fall into environmental space outside the range represented in the calibration dataset, which may reduce the reliability of fine-scale local suitability estimates [61]. Accordingly, the future distribution projections presented here primarily reflect the overall trend of shifts in climatically suitable habitats for the two species under future climate change, rather than precise predictions of fine-scale distribution changes, and their interpretation should be constrained to the scope of the above limitations. Future studies incorporating multimodel climate ensembles, dynamic future human activity scenarios, higher-resolution climate data, explicit consideration of biotic interactions, spatially structured validation frameworks, and optimized modeling frameworks will further improve the robustness and accuracy of species distribution projections.

Habitat suitability predictions are validated by documented capture records, with *P. okadai* captured across China, except Tibet, [62] and *P. variegata* recorded across the UK, France, Belgium, the Netherlands, Italy, Sweden, the European part of Turkey, and even the USA (Supplementary Fig. S1)—with virtually the entire Mediterranean coastal belt and its offshore islands falling firmly within its suitable range (Fig. 7B) [4, 7, 43, 53], aligning with model projections. Future scenarios reveal contrasting trajectories: *P. okadai* shows a projected increase in climatically suitable habitats in both China and Europe, with poleward shifts under climate warming showing a strong correlation with changes in Bio 18, which is hypothesized to be a key factor influencing the species’ range shift [63]. Conversely, *P. variegata* exhibits a distinct redistribution of its climatically suitable habitats: The transitional belt linking Central, Southern, and Eastern Europe is projected to lose virtually all suitable habitat across all scenarios, whereas the Mediterranean littoral and its offshore islands remain persistently climatically suitable.

This distribution and future suitability pattern for *P. variegata* is further corroborated by the recently frequent occurrence of *T. callipaeda* cases in the Mediterranean region, which confirms the high habitat suitability of this area for the species as the primary vector of the parasite [64]. Importantly, projections reflect climatic carrying capacity rather than actual distribution, as they do not account for nonclimatic factors critical to establishment—including dispersal ability, host availability, and repeated introductions (e.g., via fruit trade [55, 56]). For instance, a single capture of *P. okadai* in Italy [9] does not confirm a self-sustaining population, and the projected 510.6–1459.9% increase in suitable habitats for *P. okadai* reflects potential habitat availability rather than colonization probability. These considerations are particularly relevant for invasion risk assessment: Surveillance should prioritize early detection of establishment at introduction sites rather than assuming inevitable invasion across all climatically suitable areas, especially in regions where compatible hosts or parasite strains may be limited.

## Conclusions

This study systematically characterized the climatic niche differentiation and climate-driven distribution dynamics of *P. okadai* and *P. variegata*, the two primary vectors of *T. callipaeda*, across their native and potential invasive ranges. Our core findings confirm that the two vectors exhibit significant species-specific environmental adaptation strategies, with diametrically opposite shift trajectories of climatically suitable habitats under future climate change: *P. okadai* shows an overall expansion trend of suitable habitats, while *P. variegata* faces notable contraction. These results provide a scientific basis for formulating region-specific surveillance and early warning and vector control strategies for thelaziasis, especially for the invasion risk assessment of *P. okadai* in Europe. The interpretation of our results is constrained by core study limitations, including uncertainties from model input data, reliance on a single climate model, and unaccounted ecological factors (e.g., biotic interactions or host availability) that modulate the actual colonization and distribution of the two vectors. Future studies incorporating more comprehensive ecological data and multiscenario multimodel simulations will further improve the robustness of distribution projections and support more precise prevention and control of vector-borne thelaziasis.

## Supplementary Information

**Additional file 1: Fig. S1.** Suitable area in the eastern United States where *P. variegata* was discovered. **Fig. S2.** Coordinates of suitable area for *P. okadai* in Italy and location of documented capture. **Table S1**. Occurrence records and data sources for *P. okadai* and *P. variegata.*

## Data Availability

All supporting data and protocols have been provided within the article or through supplementary data files.

## Abbreviations

SDM: Species distribution modeling
MaxEnt: Maximum entropy
GBIF: Global Biodiversity Information Facility
ENMTools: Ecological niche modeling tools
AUC-ROC: Area under the receiver operating characteristic curve
TSS: True skill statistic
BI: Boyce index
SSP: Shared Socioeconomic Pathway
HFP: Human Footprint Index
