# Supplementary material for: Contrasting shifts in potential climatic suitability of two Thelazia callipaeda vectors (Phortica okadai and Phortica variegata) across China and Europe under climate change
Source: Parasit Vectors. 2026 Apr 24;19:242. doi: 10.1186/s13071-026-07417-x (PMC13244833; doi:10.1186/s13071-026-07417-x)
Supplement: Supplementary file 1 — Additional file 1: Fig. S1. Suitable area in the eastern United States where P. variegata was discovered. Fig. S2. Coordinates of suitable area for P. okadai in Italy and location of documented capture. Table S1. Occurrence records and data sources for P. okadai and P. variegata. [file 13071_2026_7417_MOESM1_ESM.docx]

**Fig. S1.** Suitable area in the eastern United States where *P. variegata* was discovered.


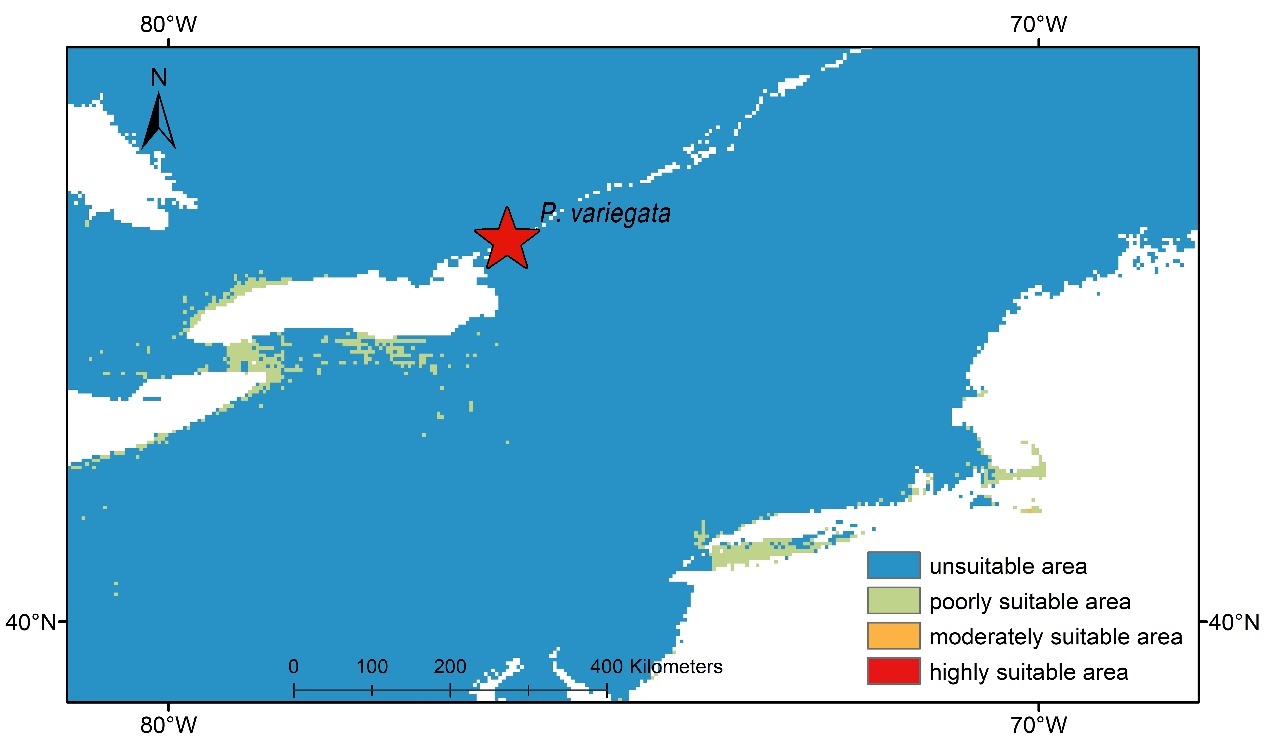


**Fig. S2.** Coordinates of suitable area for *P. okadai* in Italy and location of documented capture.


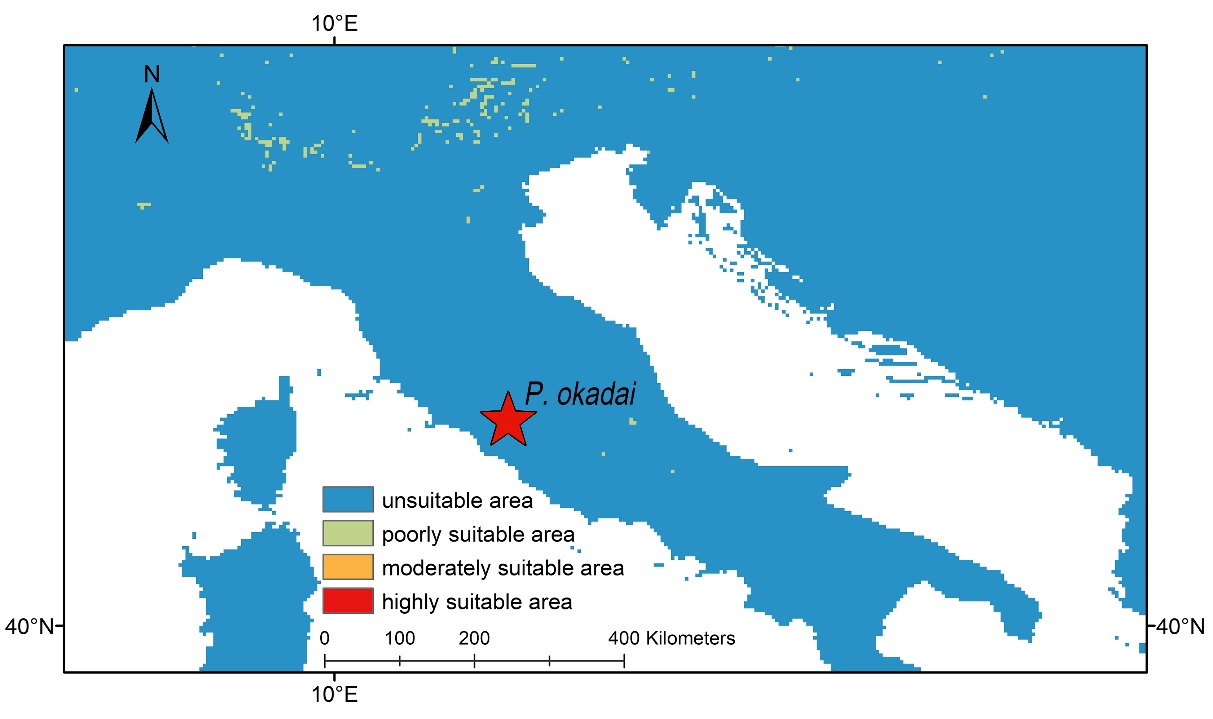


**Table S1.** Occurrence records and data sources for *P. okadai* and *P. variegata.*

| **Species** | **Number** | **Latitude** | **Longitude** | **Country or region** | **Spatial uncertainty** | **Location** | **Source of coordinates** |
| --- | --- | --- | --- | --- | --- | --- | --- |
| *P. okadai* | 1 | 39.72 | 116.71 | China | ≤100 m | Tongzhou District, Beijing, Beijing Municipality | 26.Yang Fan YF, Wang Zehua WZ, Sun Ang SA, Wang Jing WJ, Fan Rong FR, Wang Shanning WS. Species composition and population dynamics of fruit flies in polyculture cherry orchard in Beijing. China Fruits. 2024;(01):108-114. |
| *P. okadai* | 2 | 31.86 | 117.27 | China | ≤100 m | Luyang District, Hefei, Anhui Province | 27.Luo QingLi LQ, Zhou YinDi ZY, Wen HuiQin WH, Wang ZengXian WZ, Shen JiLong SJ. An investigation on the ecological character of *Amiota* (*Phortica*) *okadai*, the intermediate host of *Thelazia callipaeda*. Chinese Journal of Zoonoses. 2008;(06):548-550. |
| *P. okadai* | 3 | 31.72 | 117.01 | China | ≤100 m | Zipeng Town, Hefei, Anhui Province |  |
| *P. okadai* | 4 | 33.13 | 117.89 | China | ≤100 m | Wuhe County, Bengbu, Anhui Province |  |
| *P. okadai* | 5 | 31.16 | 118.21 | China | ≤100 m | Fanchang District, Wuhu, Anhui Province |  |
| *P. okadai* | 6 | 32.39 | 111.68 | China | ≤100 m | Laohekou City, Xiangyang, Hubei Province | 28.Wang ZX, Hu Y, Shen JL, Wang KC, Wang HY, Jiang BL, Zhao P, Wang ZC, Ding W, Wang F, Xia XF. Longitudinal investigation and experimental studies on thelaziasis and the intermediate host of *Thelazia callipaeda* in Guanghua county of Hubei province. Chinese Journal of Epidemiology. 2003;24(7):588-90. |
| *P. okadai* | 7 | 31.83 | 117.23 | China | ≤100 m | Shushan District, Hefei, Anhui Province | 29. Wang ZX, Wang CL, Shen JL, Du JS, Jiang BL, Wang HY, et al. Investigations on the epidemiological factors of thelaziasis in Jiangxi province of China. Acta Universitatis Medicinalis Anhui. 2004;39(5):364-366. |
| *P. okadai* | 8 | 29.27 | 117.18 | China | ≤100 m | Changjiang District, Jingdezhen, Jiangxi Province |  |
| *P. okadai* | 9 | 32.17 | 115.66 | China | ≤35 km | Gushi County, Xinyang, Henan Province | 30.Wang ZX, Shen JL, Wang HY, Otranto D. An update on the research of human thelaziosis. Chinese Journal of Parasitology & Parasitic Diseases. 2006;24(4):299-303. |
| *P. okadai* | 10 | 39.54 | 116.69 | China | ≤35 km | Guangyang District, Langfang, Hebei Province |  |
| *P. okadai* | 11 | 30.15 | 118.17 | China | ≤35 km | Huangshan District, Huangshan, Anhui Province | 31. Shen JL, Wang ZX, Luo QL, Li J, Wen HQ, Zhou YD. *Amiota magna* as an intermediate host of *Thelazia callipaeda* under laboratory conditions. Chinese Journal of Parasitology & Parasitic Diseases. 2009;27(4):375-6. |
| *P. okadai* | 12 | 30.08 | 118.19 | China | ≤35 km | Huangshan District, Huangshan, Anhui Province |  |
| *P. okadai* | 13 | 33.96 | 116.80 | China | ≤100 m | Xiangshan District, Huaibei, Anhui Province | 32. Wang HY, Wang ZX, Wang KC, Hu Y, Shen JL, Niu JH, et al. Studies on the relationship between human thelaziasis and canine *Thelazia callipaeda* infection. International Eye Science. 2010;10(11):2086-2087. |
| *P. okadai* | 14 | 31.11 | 115.78 | China | ≤100 m | Tiantangzhai Town, Lu'an, Anhui Province | 33. Xie LC. Investigation of Chinese human thelaziasis. Journal of Pathogen Biology. 2010;5(08):630-632+641. |
| *P. okadai* | 15 | 31.89 | 117.09 | China | ≤100 m | Shushan District, Hefei, Anhui Province | 34. Zhu L. The Taxonomy of Genus *Phortica* (Diptera: Drosophilidae) from China, with DNA Barcoding. South China Agricultural University. 2017. |
| *P. okadai* | 16 | 30.16 | 114.48 | China | ≤100 m | Jiangxia Liangzihu District, Wuhan, Hubei Province | 35. Jing Z, Yuanhuai Q, Xiaoqiao X. The first report of study of karyotype on *Amiota* *Okadai*. Journal of Hubei University (natural Science). 1998;20(3):277-9. |
| *P. okadai* | 17 | 30.58 | 114.34 | China | ≤35 km | Wuchang District, Wuhan, Hubei Province |  |
| *P. okadai* | 18 | 31.26 | 117.88 | China | ≤35 km | Shilidun Town, Wuwei, Wuhu, Anhui Province | 36. Wang ZX, Shen JL, Du JH, Jiang BL, Han XQ, Wang ZH. Studies on the relationship between alternation of generations or dogs and transmission of thelaziasis. Journal of Tropical Diseases and Parasitology. 2003;(04):204-207. |
| *P. okadai* | 19 | 27.77 | 106.93 | China | ≤100 m | Huichuan District, Zunyi, Guizhou Province | 37. Huang XG, Zhang LF, Wang LJ, Zheng MH, Liu H. The zoophilic fruitfly *Amiota okadai* in Zunyi City:flies capture, morphology identification and laboratory breeding. Journal of Medical Pest Control. 2017;33(07):765-766+770. |
| *P. okadai* | 20 | 27.68 | 107.01 | China | ≤100 m | Bozhou District, Zunyi, Guizhou Province |  |
| *P. okadai* | 21 | 27.61 | 106.90 | China | ≤100 m | Honghuagang District, Zunyi, Guizhou Province |  |
| *P. okadai* | 22 | 33.72 | 107.98 | China | ≤100 m | Foping County, Hanzhong, Shaanxi Province | the Global Biodiversity Information Facility (GBIF; https://www.gbif.org/) |
| *P. okadai* | 23 | 26.10 | 105.89 | China | ≤100 m | Xinchang Buyi and Miao Ethnic Township, Anshun, Guizhou Province |  |
| *P. okadai* | 24 | 30.33 | 119.42 | China | ≤100 m | Lin'an District, Hangzhou, Zhejiang Province |  |
| *P. okadai* | 25 | 30.24 | 119.73 | China | ≤100 m | Lin'an District, Hangzhou, Zhejiang Province |  |
| *P. okadai* | 26 | 29.27 | 117.85 | China | ≤100 m | Wuyuan County, Shangrao, Jiangxi Province |  |
| *P. okadai* | 27 | 37.26 | 121.72 | China | ≤100 m | Muping District, Yantai, Shandong Province |  |
| *P. okadai* | 28 | 41.84 | 123.59 | China | ≤100 m | Hunnan District, Shenyang, Liaoning Province |  |
| *P. okadai* | 29 | 41.13 | 124.16 | China | ≤100 m | Caohezhang Town, Benxi, Liaoning Province |  |
| *P. okadai* | 30 | 27.85 | 107.26 | China | ≤100 m | Honghuagang District, Zunyi, Guizhou Province | The findings of the field investigation conducted by the author's research group in the Zunyi area |
| *P. variegata* | 1 | 56.26 | 14.97 | Europe | ≤100 m | Karlshamn, Blekinge County, Sweden | the Global Biodiversity Information Facility (GBIF; https://www.gbif.org/) |
| *P. variegata* | 2 | 57.03 | 16.14 | Europe | ≤100 m | Högsby, Kalmar County, Sweden | the Global Biodiversity Information Facility (GBIF; https://www.gbif.org/) |
| *P. variegata* | 3 | 55.60 | 13.42 | Europe | ≤100 m | Lund, Skåne County, Sweden | the Global Biodiversity Information Facility (GBIF; https://www.gbif.org/) |
| *P. variegata* | 4 | 50.83 | -1.50 | Europe | ≤100 m | Hampshire, England, United Kingdom | the Global Biodiversity Information Facility (GBIF; https://www.gbif.org/) |
| *P. variegata* | 5 | 51.81 | -0.59 | Europe | ≤100 m | Buckinghamshire, England, United Kingdom | the Global Biodiversity Information Facility (GBIF; https://www.gbif.org/) |
| *P. variegata* | 6 | 48.39 | 2.67 | Europe | ≤100 m | Seine-et-Marne, Île-de-France, France | the Global Biodiversity Information Facility (GBIF; https://www.gbif.org/) |
| *P. variegata* | 7 | 44.61 | 1.17 | Europe | ≤100 m | Lot, Occitanie, France | the Global Biodiversity Information Facility (GBIF; https://www.gbif.org/) |
| *P. variegata* | 8 | 44.61 | 1.17 | Europe | ≤100 m | Lot, Occitanie, France | the Global Biodiversity Information Facility (GBIF; https://www.gbif.org/) |
| *P. variegata* | 9 | 57.86 | 15.44 | Europe | ≤100 m | Ydre, Östergötland County, Sweden | the Global Biodiversity Information Facility (GBIF; https://www.gbif.org/) |
| *P. variegata* | 10 | 57.85 | 15.53 | Europe | ≤100 m | Kinda, Östergötland County, Sweden | the Global Biodiversity Information Facility (GBIF; https://www.gbif.org/) |
| *P. variegata* | 11 | 57.85 | 15.53 | Europe | ≤100 m | Kinda, Östergötland County, Sweden | the Global Biodiversity Information Facility (GBIF; https://www.gbif.org/) |
| *P. variegata* | 12 | 58.38 | 15.56 | Europe | ≤100 m | Linköping, Östergötland County, Sweden | the Global Biodiversity Information Facility (GBIF; https://www.gbif.org/) |
| *P. variegata* | 13 | 58.38 | 15.56 | Europe | ≤100 m | Linköping, Östergötland County, Sweden | the Global Biodiversity Information Facility (GBIF; https://www.gbif.org/) |
| *P. variegata* | 14 | 58.15 | 15.72 | Europe | ≤100 m | Kinda, Östergötland County, Sweden | the Global Biodiversity Information Facility (GBIF; https://www.gbif.org/) |
| *P. variegata* | 15 | 57.86 | 15.44 | Europe | ≤100 m | Ydre, Östergötland County, Sweden | the Global Biodiversity Information Facility (GBIF; https://www.gbif.org/) |
| *P. variegata* | 16 | 58.38 | 15.56 | Europe | ≤100 m | Linköping, Östergötland County, Sweden | the Global Biodiversity Information Facility (GBIF; https://www.gbif.org/) |
| *P. variegata* | 17 | 58.38 | 15.56 | Europe | ≤100 m | Linköping, Östergötland County, Sweden | the Global Biodiversity Information Facility (GBIF; https://www.gbif.org/) |
| *P. variegata* | 18 | 57.85 | 15.53 | Europe | ≤100 m | Kinda, Östergötland County, Sweden | the Global Biodiversity Information Facility (GBIF; https://www.gbif.org/) |
| *P. variegata* | 19 | 57.87 | 15.44 | Europe | ≤100 m | Ydre, Östergötland County, Sweden | the Global Biodiversity Information Facility (GBIF; https://www.gbif.org/) |
| *P. variegata* | 20 | 58.15 | 15.72 | Europe | ≤100 m | Kinda, Östergötland County, Sweden | the Global Biodiversity Information Facility (GBIF; https://www.gbif.org/) |
| *P. variegata* | 21 | 57.85 | 15.53 | Europe | ≤100 m | Kinda, Östergötland County, Sweden | the Global Biodiversity Information Facility (GBIF; https://www.gbif.org/) |
| *P. variegata* | 22 | 58.38 | 15.56 | Europe | ≤100 m | Linköping, Östergötland County, Sweden | the Global Biodiversity Information Facility (GBIF; https://www.gbif.org/) |
| *P. variegata* | 23 | 58.38 | 15.56 | Europe | ≤100 m | Linköping, Östergötland County, Sweden | the Global Biodiversity Information Facility (GBIF; https://www.gbif.org/) |
| *P. variegata* | 24 | 57.86 | 15.44 | Europe | ≤100 m | Ydre, Östergötland County, Sweden | the Global Biodiversity Information Facility (GBIF; https://www.gbif.org/) |
| *P. variegata* | 25 | 58.38 | 15.56 | Europe | ≤100 m | Linköping, Östergötland County, Sweden | the Global Biodiversity Information Facility (GBIF; https://www.gbif.org/) |
| *P. variegata* | 26 | 58.38 | 15.56 | Europe | ≤100 m | Linköping, Östergötland County, Sweden | the Global Biodiversity Information Facility (GBIF; https://www.gbif.org/) |
| *P. variegata* | 27 | 58.38 | 15.56 | Europe | ≤100 m | Linköping, Östergötland County, Sweden | the Global Biodiversity Information Facility (GBIF; https://www.gbif.org/) |
| *P. variegata* | 28 | 58.38 | 15.56 | Europe | ≤100 m | Linköping, Östergötland County, Sweden | the Global Biodiversity Information Facility (GBIF; https://www.gbif.org/) |
| *P. variegata* | 29 | 58.38 | 15.56 | Europe | ≤100 m | Linköping, Östergötland County, Sweden | the Global Biodiversity Information Facility (GBIF; https://www.gbif.org/) |
| *P. variegata* | 30 | 58.38 | 15.56 | Europe | ≤100 m | Linköping, Östergötland County, Sweden | the Global Biodiversity Information Facility (GBIF; https://www.gbif.org/) |
| *P. variegata* | 31 | 58.38 | 15.56 | Europe | ≤100 m | Linköping, Östergötland County, Sweden | the Global Biodiversity Information Facility (GBIF; https://www.gbif.org/) |
| *P. variegata* | 32 | 58.38 | 15.56 | Europe | ≤100 m | Karlshamn, Blekinge County, Sweden | the Global Biodiversity Information Facility (GBIF; https://www.gbif.org/) |
| *P. variegata* | 33 | 58.38 | 15.56 | Europe | ≤100 m | Högsby, Kalmar County, Sweden | the Global Biodiversity Information Facility (GBIF; https://www.gbif.org/) |
| *P. variegata* | 34 | 58.38 | 15.56 | Europe | ≤100 m | Lund, Skåne County, Sweden | the Global Biodiversity Information Facility (GBIF; https://www.gbif.org/) |
| *P. variegata* | 35 | 58.38 | 15.56 | Europe | ≤100 m | Hampshire, England, United Kingdom | the Global Biodiversity Information Facility (GBIF; https://www.gbif.org/) |
| *P. variegata* | 36 | 58.38 | 15.56 | Europe | ≤100 m | Buckinghamshire, England, United Kingdom | the Global Biodiversity Information Facility (GBIF; https://www.gbif.org/) |
| *P. variegata* | 37 | 58.15 | 15.72 | Europe | ≤100 m | Seine-et-Marne, Île-de-France, France | the Global Biodiversity Information Facility (GBIF; https://www.gbif.org/) |
| *P. variegata* | 38 | 58.15 | 15.72 | Europe | ≤100 m | Lot, Occitanie, France | the Global Biodiversity Information Facility (GBIF; https://www.gbif.org/) |
| *P. variegata* | 39 | 58.38 | 15.56 | Europe | ≤100 m | Lot, Occitanie, France | the Global Biodiversity Information Facility (GBIF; https://www.gbif.org/) |
| *P. variegata* | 40 | 58.15 | 15.72 | Europe | ≤100 m | Ydre, Östergötland County, Sweden | the Global Biodiversity Information Facility (GBIF; https://www.gbif.org/) |
| *P. variegata* | 41 | 57.84 | 18.60 | Europe | ≤100 m | Kinda, Östergötland County, Sweden | the Global Biodiversity Information Facility (GBIF; https://www.gbif.org/) |
| *P. variegata* | 42 | 36.89 | -2.61 | Europe | ≤100 m | Kinda, Östergötland County, Sweden | the Global Biodiversity Information Facility (GBIF; https://www.gbif.org/) |
| *P. variegata* | 43 | 59.86 | 18.60 | Europe | ≤100 m | Linköping, Östergötland County, Sweden | the Global Biodiversity Information Facility (GBIF; https://www.gbif.org/) |
| *P. variegata* | 44 | 58.20 | 14.91 | Europe | ≤100 m | Linköping, Östergötland County, Sweden | the Global Biodiversity Information Facility (GBIF; https://www.gbif.org/) |
| *P. variegata* | 45 | 51.67 | -2.68 | Europe | ≤100 m | Kinda, Östergötland County, Sweden | the Global Biodiversity Information Facility (GBIF; https://www.gbif.org/) |
| *P. variegata* | 46 | 50.85 | 5.75 | Europe | ≤100 m | Ydre, Östergötland County, Sweden | the Global Biodiversity Information Facility (GBIF; https://www.gbif.org/) |
| *P. variegata* | 47 | 56.79 | 16.56 | Europe | ≤100 m | Linköping, Östergötland County, Sweden | the Global Biodiversity Information Facility (GBIF; https://www.gbif.org/) |
| *P. variegata* | 48 | 48.16 | 16.22 | Europe | ≤100 m | Linköping, Östergötland County, Sweden | the Global Biodiversity Information Facility (GBIF; https://www.gbif.org/) |
| *P. variegata* | 49 | 44.28 | 4.56 | Europe | ≤100 m | Kinda, Östergötland County, Sweden | the Global Biodiversity Information Facility (GBIF; https://www.gbif.org/) |
| *P. variegata* | 50 | 48.39 | 2.67 | Europe | ≤100 m | Ydre, Östergötland County, Sweden | the Global Biodiversity Information Facility (GBIF; https://www.gbif.org/) |
| *P. variegata* | 51 | 44.28 | 4.56 | Europe | ≤100 m | Kinda, Östergötland County, Sweden | the Global Biodiversity Information Facility (GBIF; https://www.gbif.org/) |
| *P. variegata* | 52 | 58.33 | 15.77 | Europe | ≤100 m | Kinda, Östergötland County, Sweden | the Global Biodiversity Information Facility (GBIF; https://www.gbif.org/) |
| *P. variegata* | 53 | 58.33 | 15.77 | Europe | ≤100 m | Linköping, Östergötland County, Sweden | the Global Biodiversity Information Facility (GBIF; https://www.gbif.org/) |
| *P. variegata* | 54 | 51.80 | -0.59 | Europe | ≤100 m | Linköping, Östergötland County, Sweden | the Global Biodiversity Information Facility (GBIF; https://www.gbif.org/) |
| *P. variegata* | 55 | 51.95 | -0.67 | Europe | ≤100 m | Ydre, Östergötland County, Sweden | the Global Biodiversity Information Facility (GBIF; https://www.gbif.org/) |
| *P. variegata* | 56 | 51.67 | 5.27 | Europe | ≤100 m | Linköping, Östergötland County, Sweden | the Global Biodiversity Information Facility (GBIF; https://www.gbif.org/) |
| *P. variegata* | 57 | 51.67 | 5.27 | Europe | ≤100 m | Linköping, Östergötland County, Sweden | the Global Biodiversity Information Facility (GBIF; https://www.gbif.org/) |
| *P. variegata* | 58 | 51.53 | 5.01 | Europe | ≤100 m | Linköping, Östergötland County, Sweden | the Global Biodiversity Information Facility (GBIF; https://www.gbif.org/) |
| *P. variegata* | 59 | 51.53 | 5.01 | Europe | ≤100 m | Linköping, Östergötland County, Sweden | the Global Biodiversity Information Facility (GBIF; https://www.gbif.org/) |
| *P. variegata* | 60 | 51.53 | 5.01 | Europe | ≤100 m | Linköping, Östergötland County, Sweden | the Global Biodiversity Information Facility (GBIF; https://www.gbif.org/) |
| *P. variegata* | 61 | 51.53 | 5.01 | Europe | ≤100 m | Linköping, Östergötland County, Sweden | the Global Biodiversity Information Facility (GBIF; https://www.gbif.org/) |
| *P. variegata* | 62 | 51.53 | 5.01 | Europe | ≤100 m | Linköping, Östergötland County, Sweden | the Global Biodiversity Information Facility (GBIF; https://www.gbif.org/) |
| *P. variegata* | 63 | 43.70 | 5.45 | Europe | ≤100 m | Vaucluse, Provence-Alpes-Côte d’Azur, France | the Global Biodiversity Information Facility (GBIF; https://www.gbif.org/) |
| *P. variegata* | 64 | 57.13 | 16.47 | Europe | ≤100 m | Oskarshamn, Kalmar County, Sweden | the Global Biodiversity Information Facility (GBIF; https://www.gbif.org/) |
| *P. variegata* | 65 | 57.13 | 16.47 | Europe | ≤100 m | Oskarshamn, Kalmar County, Sweden | the Global Biodiversity Information Facility (GBIF; https://www.gbif.org/) |
| *P. variegata* | 66 | 57.12 | 16.35 | Europe | ≤100 m | Mönsterås, Kalmar County, Sweden | the Global Biodiversity Information Facility (GBIF; https://www.gbif.org/) |
| *P. variegata* | 67 | 57.13 | 16.47 | Europe | ≤100 m | Oskarshamn, Kalmar County, Sweden | the Global Biodiversity Information Facility (GBIF; https://www.gbif.org/) |
| *P. variegata* | 68 | 57.12 | 16.35 | Europe | ≤100 m | Mönsterås, Kalmar County, Sweden | the Global Biodiversity Information Facility (GBIF; https://www.gbif.org/) |
| *P. variegata* | 69 | 56.81 | 15.91 | Europe | ≤100 m | Nybro, Kalmar County, Sweden | the Global Biodiversity Information Facility (GBIF; https://www.gbif.org/) |
| *P. variegata* | 70 | 56.81 | 15.89 | Europe | ≤100 m | Nybro, Kalmar County, Sweden | the Global Biodiversity Information Facility (GBIF; https://www.gbif.org/) |
| *P. variegata* | 71 | 56.81 | 15.89 | Europe | ≤100 m | Nybro, Kalmar County, Sweden | the Global Biodiversity Information Facility (GBIF; https://www.gbif.org/) |
| *P. variegata* | 72 | 57.12 | 16.35 | Europe | ≤100 m | Mönsterås, Kalmar County, Sweden | the Global Biodiversity Information Facility (GBIF; https://www.gbif.org/) |
| *P. variegata* | 73 | 57.12 | 16.35 | Europe | ≤100 m | Mönsterås, Kalmar County, Sweden | the Global Biodiversity Information Facility (GBIF; https://www.gbif.org/) |
| *P. variegata* | 74 | 56.81 | 15.89 | Europe | ≤100 m | Nybro, Kalmar County, Sweden | the Global Biodiversity Information Facility (GBIF; https://www.gbif.org/) |
| *P. variegata* | 75 | 57.52 | 16.47 | Europe | ≤100 m | Västervik, Kalmar County, Sweden | the Global Biodiversity Information Facility (GBIF; https://www.gbif.org/) |
| *P. variegata* | 76 | 57.12 | 16.35 | Europe | ≤100 m | Mönsterås, Kalmar County, Sweden | the Global Biodiversity Information Facility (GBIF; https://www.gbif.org/) |
| *P. variegata* | 77 | 56.81 | 15.89 | Europe | ≤100 m | Nybro, Kalmar County, Sweden | the Global Biodiversity Information Facility (GBIF; https://www.gbif.org/) |
| *P. variegata* | 78 | 58.33 | 15.71 | Europe | ≤100 m | Linköping, Östergötland County, Sweden | the Global Biodiversity Information Facility (GBIF; https://www.gbif.org/) |
| *P. variegata* | 79 | 58.33 | 15.72 | Europe | ≤100 m | Linköping, Östergötland County, Sweden | the Global Biodiversity Information Facility (GBIF; https://www.gbif.org/) |
| *P. variegata* | 80 | 58.33 | 15.72 | Europe | ≤100 m | Linköping, Östergötland County, Sweden | the Global Biodiversity Information Facility (GBIF; https://www.gbif.org/) |
| *P. variegata* | 81 | 58.32 | 15.72 | Europe | ≤100 m | Linköping, Östergötland County, Sweden | the Global Biodiversity Information Facility (GBIF; https://www.gbif.org/) |
| *P. variegata* | 82 | 58.32 | 15.72 | Europe | ≤100 m | Linköping, Östergötland County, Sweden | the Global Biodiversity Information Facility (GBIF; https://www.gbif.org/) |
| *P. variegata* | 83 | 58.39 | 15.61 | Europe | ≤100 m | Linköping, Östergötland County, Sweden | the Global Biodiversity Information Facility (GBIF; https://www.gbif.org/) |
| *P. variegata* | 84 | 58.39 | 15.61 | Europe | ≤100 m | Linköping, Östergötland County, Sweden | the Global Biodiversity Information Facility (GBIF; https://www.gbif.org/) |
| *P. variegata* | 85 | 49.72 | 31.53 | Europe | ≤100 m | Kanivs’kyi District, Cherkasy Oblast, Ukraine | the Global Biodiversity Information Facility (GBIF; https://www.gbif.org/) |
| *P. variegata* | 86 | 57.26 | 15.91 | Europe | ≤100 m | Hultsfred, Kalmar County, Sweden | the Global Biodiversity Information Facility (GBIF; https://www.gbif.org/) |
| *P. variegata* | 87 | 58.32 | 14.68 | Europe | ≤100 m | Ödeshög, Östergötland County, Sweden | the Global Biodiversity Information Facility (GBIF; https://www.gbif.org/) |
| *P. variegata* | 88 | 58.32 | 14.68 | Europe | ≤100 m | Ödeshög, Östergötland County, Sweden | the Global Biodiversity Information Facility (GBIF; https://www.gbif.org/) |
| *P. variegata* | 89 | 58.34 | 14.65 | Europe | ≤100 m | Ödeshög, Östergötland County, Sweden | the Global Biodiversity Information Facility (GBIF; https://www.gbif.org/) |
| *P. variegata* | 90 | 58.34 | 14.65 | Europe | ≤100 m | Ödeshög, Östergötland County, Sweden | the Global Biodiversity Information Facility (GBIF; https://www.gbif.org/) |
| *P. variegata* | 91 | 58.34 | 14.65 | Europe | ≤100 m | Ödeshög, Östergötland County, Sweden | the Global Biodiversity Information Facility (GBIF; https://www.gbif.org/) |
| *P. variegata* | 92 | 58.34 | 14.65 | Europe | ≤100 m | Ödeshög, Östergötland County, Sweden | the Global Biodiversity Information Facility (GBIF; https://www.gbif.org/) |
| *P. variegata* | 93 | 58.34 | 14.65 | Europe | ≤100 m | Ödeshög, Östergötland County, Sweden | the Global Biodiversity Information Facility (GBIF; https://www.gbif.org/) |
| *P. variegata* | 94 | 46.13 | 5.25 | Europe | ≤100 m | Ain, Auvergne-Rhône-Alpes, France | the Global Biodiversity Information Facility (GBIF; https://www.gbif.org/) |
| *P. variegata* | 95 | 46.02 | 4.54 | Europe | ≤100 m | Rhône, Auvergne-Rhône-Alpes, France | the Global Biodiversity Information Facility (GBIF; https://www.gbif.org/) |
| *P. variegata* | 96 | 60.38 | 23.14 | Europe | ≤100 m | Varsinais-Suomi, Western Finland, Finland | the Global Biodiversity Information Facility (GBIF; https://www.gbif.org/) |
| *P. variegata* | 97 | 60.40 | 23.10 | Europe | ≤100 m | Varsinais-Suomi, Western Finland, Finland | the Global Biodiversity Information Facility (GBIF; https://www.gbif.org/) |
| *P. variegata* | 98 | 58.34 | 14.66 | Europe | ≤100 m | Ödeshög, Östergötland County, Sweden | the Global Biodiversity Information Facility (GBIF; https://www.gbif.org/) |
| *P. variegata* | 99 | 58.30 | 14.65 | Europe | ≤100 m | Ödeshög, Östergötland County, Sweden | the Global Biodiversity Information Facility (GBIF; https://www.gbif.org/) |
| *P. variegata* | 100 | 58.30 | 14.64 | Europe | ≤100 m | Ödeshög, Östergötland County, Sweden | the Global Biodiversity Information Facility (GBIF; https://www.gbif.org/) |
| *P. variegata* | 101 | 58.35 | 14.69 | Europe | ≤100 m | Vadstena, Östergötland County, Sweden | the Global Biodiversity Information Facility (GBIF; https://www.gbif.org/) |
| *P. variegata* | 102 | 58.30 | 14.64 | Europe | ≤100 m | Ödeshög, Östergötland County, Sweden | the Global Biodiversity Information Facility (GBIF; https://www.gbif.org/) |
| *P. variegata* | 103 | 58.34 | 14.65 | Europe | ≤100 m | Ödeshög, Östergötland County, Sweden | the Global Biodiversity Information Facility (GBIF; https://www.gbif.org/) |
| *P. variegata* | 104 | 41.18 | -8.48 | Europe | ≤100 m | Valongo, Porto District, Portugal | the Global Biodiversity Information Facility (GBIF; https://www.gbif.org/) |
| *P. variegata* | 105 | 51.89 | 0.43 | Europe | ≤100 m | Essex, England, United Kingdom | the Global Biodiversity Information Facility (GBIF; https://www.gbif.org/) |
| *P. variegata* | 106 | 51.47 | -1.16 | Europe | ≤100 m | Berkshire, England, United Kingdom | the Global Biodiversity Information Facility (GBIF; https://www.gbif.org/) |
| *P. variegata* | 107 | 51.37 | -0.25 | Europe | ≤100 m | Surrey, England, United Kingdom | the Global Biodiversity Information Facility (GBIF; https://www.gbif.org/) |
| *P. variegata* | 108 | 58.25 | 15.67 | Europe | ≤100 m | Linköping, Östergötland County, Sweden | the Global Biodiversity Information Facility (GBIF; https://www.gbif.org/) |
| *P. variegata* | 109 | 56.13 | 12.69 | Europe | ≤100 m | Helsingborg, Skåne County, Sweden | the Global Biodiversity Information Facility (GBIF; https://www.gbif.org/) |
| *P. variegata* | 110 | 51.95 | 5.85 | Europe | ≤100 m | Arnhem, Gelderland, Netherlands | the Global Biodiversity Information Facility (GBIF; https://www.gbif.org/) |
| *P. variegata* | 111 | 51.95 | 5.85 | Europe | ≤100 m | Arnhem, Gelderland, Netherlands | the Global Biodiversity Information Facility (GBIF; https://www.gbif.org/) |
| *P. variegata* | 112 | 51.95 | 5.85 | Europe | ≤100 m | Arnhem, Gelderland, Netherlands | the Global Biodiversity Information Facility (GBIF; https://www.gbif.org/) |
| *P. variegata* | 113 | 50.80 | 5.75 | Europe | ≤100 m | Eijsden, Limburg, Netherlands | the Global Biodiversity Information Facility (GBIF; https://www.gbif.org/) |
| *P. variegata* | 114 | 41.18 | -8.48 | Europe | ≤100 m | Valongo, Porto District, Portugal | the Global Biodiversity Information Facility (GBIF; https://www.gbif.org/) |
| *P. variegata* | 115 | 41.18 | -8.48 | Europe | ≤100 m | Valongo, Porto District, Portugal | the Global Biodiversity Information Facility (GBIF; https://www.gbif.org/) |
| *P. variegata* | 116 | 57.86 | 16.53 | Europe | ≤100 m | Västervik, Kalmar County, Sweden | the Global Biodiversity Information Facility (GBIF; https://www.gbif.org/) |
| *P. variegata* | 117 | 56.99 | 16.03 | Europe | ≤100 m | Högsby, Kalmar County, Sweden | the Global Biodiversity Information Facility (GBIF; https://www.gbif.org/) |
| *P. variegata* | 118 | 57.86 | 16.53 | Europe | ≤100 m | Västervik, Kalmar County, Sweden | the Global Biodiversity Information Facility (GBIF; https://www.gbif.org/) |
| *P. variegata* | 119 | 56.99 | 16.03 | Europe | ≤100 m | Högsby, Kalmar County, Sweden | the Global Biodiversity Information Facility (GBIF; https://www.gbif.org/) |
| *P. variegata* | 120 | 56.62 | 16.51 | Europe | ≤100 m | Mörbylånga, Kalmar County, Sweden | the Global Biodiversity Information Facility (GBIF; https://www.gbif.org/) |
| *P. variegata* | 121 | 43.71 | 5.43 | Europe | ≤100 m | Vaucluse, Provence-Alpes-Côte d'Azur, France | the Global Biodiversity Information Facility (GBIF; https://www.gbif.org/) |
| *P. variegata* | 122 | 43.71 | 5.43 | Europe | ≤100 m | Vaucluse, Provence-Alpes-Côte d'Azur, France | the Global Biodiversity Information Facility (GBIF; https://www.gbif.org/) |
| *P. variegata* | 123 | 43.71 | 5.43 | Europe | ≤100 m | Vaucluse, Provence-Alpes-Côte d'Azur, France | the Global Biodiversity Information Facility (GBIF; https://www.gbif.org/) |
| *P. variegata* | 124 | 55.83 | 13.33 | Europe | ≤100 m | Eslöv, Skåne County, Sweden | the Global Biodiversity Information Facility (GBIF; https://www.gbif.org/) |
| *P. variegata* | 125 | 55.83 | 13.33 | Europe | ≤100 m | Eslöv, Skåne County, Sweden | the Global Biodiversity Information Facility (GBIF; https://www.gbif.org/) |
| *P. variegata* | 126 | 61.07 | 28.76 | Europe | ≤100 m | Vyborg District, Leningrad Oblast, Russia | the Global Biodiversity Information Facility (GBIF; https://www.gbif.org/) |
| *P. variegata* | 127 | 52.75 | 5.80 | Europe | ≤100 m | Noordoostpolder, Flevoland, Netherlands | the Global Biodiversity Information Facility (GBIF; https://www.gbif.org/) |
| *P. variegata* | 128 | 51.95 | 5.65 | Europe | ≤100 m | Wageningen, Gelderland, Netherlands | the Global Biodiversity Information Facility (GBIF; https://www.gbif.org/) |
| *P. variegata* | 129 | 37.78 | -25.32 | Europe | ≤100 m | Povoação, Azores, Portugal | the Global Biodiversity Information Facility (GBIF; https://www.gbif.org/) |
| *P. variegata* | 130 | 37.78 | -25.31 | Europe | ≤100 m | Povoação, Azores, Portugal | the Global Biodiversity Information Facility (GBIF; https://www.gbif.org/) |
| *P. variegata* | 131 | 37.77 | -25.31 | Europe | ≤100 m | Povoação, Azores, Portugal | the Global Biodiversity Information Facility (GBIF; https://www.gbif.org/) |
| *P. variegata* | 132 | 37.78 | -25.32 | Europe | ≤100 m | Povoação, Azores, Portugal | the Global Biodiversity Information Facility (GBIF; https://www.gbif.org/) |
| *P. variegata* | 133 | 37.77 | -25.32 | Europe | ≤100 m | Povoação, Azores, Portugal | the Global Biodiversity Information Facility (GBIF; https://www.gbif.org/) |
| *P. variegata* | 134 | 37.77 | -25.32 | Europe | ≤100 m | Povoação, Azores, Portugal | the Global Biodiversity Information Facility (GBIF; https://www.gbif.org/) |
| *P. variegata* | 135 | 37.77 | -25.31 | Europe | ≤100 m | Povoação, Azores, Portugal | the Global Biodiversity Information Facility (GBIF; https://www.gbif.org/) |
| *P. variegata* | 136 | 37.77 | -25.31 | Europe | ≤100 m | Povoação, Azores, Portugal | the Global Biodiversity Information Facility (GBIF; https://www.gbif.org/) |
| *P. variegata* | 137 | 37.78 | -25.31 | Europe | ≤100 m | Povoação, Azores, Portugal | the Global Biodiversity Information Facility (GBIF; https://www.gbif.org/) |
| *P. variegata* | 138 | 37.77 | -25.32 | Europe | ≤100 m | Povoação, Azores, Portugal | the Global Biodiversity Information Facility (GBIF; https://www.gbif.org/) |
| *P. variegata* | 139 | 37.77 | -25.31 | Europe | ≤100 m | Povoação, Azores, Portugal | the Global Biodiversity Information Facility (GBIF; https://www.gbif.org/) |
| *P. variegata* | 140 | 37.78 | -25.32 | Europe | ≤100 m | Povoação, Azores, Portugal | the Global Biodiversity Information Facility (GBIF; https://www.gbif.org/) |
| *P. variegata* | 141 | 37.78 | -25.31 | Europe | ≤100 m | Povoação, Azores, Portugal | the Global Biodiversity Information Facility (GBIF; https://www.gbif.org/) |
| *P. variegata* | 142 | 37.78 | -25.31 | Europe | ≤100 m | Povoação, Azores, Portugal | the Global Biodiversity Information Facility (GBIF; https://www.gbif.org/) |
| *P. variegata* | 143 | 37.78 | -25.32 | Europe | ≤100 m | Povoação, Azores, Portugal | the Global Biodiversity Information Facility (GBIF; https://www.gbif.org/) |
| *P. variegata* | 144 | 37.77 | -25.32 | Europe | ≤100 m | Povoação, Azores, Portugal | the Global Biodiversity Information Facility (GBIF; https://www.gbif.org/) |
| *P. variegata* | 145 | 56.69 | 16.63 | Europe | ≤100 m | Povoação, Azores, Portugal | the Global Biodiversity Information Facility (GBIF; https://www.gbif.org/) |
| *P. variegata* | 146 | 56.69 | 16.63 | Europe | ≤100 m | Borgholm, Kalmar County, Sweden | the Global Biodiversity Information Facility (GBIF; https://www.gbif.org/) |
| *P. variegata* | 147 | 56.70 | 16.62 | Europe | ≤100 m | Borgholm, Kalmar County, Sweden | the Global Biodiversity Information Facility (GBIF; https://www.gbif.org/) |
| *P. variegata* | 148 | 56.69 | 16.63 | Europe | ≤100 m | Mörbylånga, Kalmar County, Sweden | the Global Biodiversity Information Facility (GBIF; https://www.gbif.org/) |
| *P. variegata* | 149 | 56.70 | 16.62 | Europe | ≤100 m | Borgholm, Kalmar County, Sweden | the Global Biodiversity Information Facility (GBIF; https://www.gbif.org/) |
| *P. variegata* | 150 | 56.16 | 15.49 | Europe | ≤100 m | Mörbylånga, Kalmar County, Sweden | the Global Biodiversity Information Facility (GBIF; https://www.gbif.org/) |
| *P. variegata* | 151 | 56.16 | 15.49 | Europe | ≤100 m | Ronneby, Blekinge County, Sweden | the Global Biodiversity Information Facility (GBIF; https://www.gbif.org/) |
| *P. variegata* | 152 | 56.17 | 15.49 | Europe | ≤100 m | Ronneby, Blekinge County, Sweden | the Global Biodiversity Information Facility (GBIF; https://www.gbif.org/) |
| *P. variegata* | 153 | 59.53 | 16.28 | Europe | ≤100 m | Ronneby, Blekinge County, Sweden | the Global Biodiversity Information Facility (GBIF; https://www.gbif.org/) |
| *P. variegata* | 154 | 56.97 | 16.02 | Europe | ≤100 m | Hallstahammar, Västmanland County, Sweden | the Global Biodiversity Information Facility (GBIF; https://www.gbif.org/) |
| *P. variegata* | 155 | 56.97 | 16.01 | Europe | ≤100 m | Nybro, Kalmar County, Sweden | the Global Biodiversity Information Facility (GBIF; https://www.gbif.org/) |
| *P. variegata* | 156 | 56.97 | 16.01 | Europe | ≤100 m | Högsby, Kalmar County, Sweden | the Global Biodiversity Information Facility (GBIF; https://www.gbif.org/) |
| *P. variegata* | 157 | 56.97 | 16.02 | Europe | ≤100 m | Högsby, Kalmar County, Sweden | the Global Biodiversity Information Facility (GBIF; https://www.gbif.org/) |
| *P. variegata* | 158 | 56.97 | 16.01 | Europe | ≤100 m | Nybro, Kalmar County, Sweden | the Global Biodiversity Information Facility (GBIF; https://www.gbif.org/) |
| *P. variegata* | 159 | 56.97 | 16.01 | Europe | ≤100 m | Högsby, Kalmar County, Sweden | the Global Biodiversity Information Facility (GBIF; https://www.gbif.org/) |
| *P. variegata* | 160 | 56.97 | 16.01 | Europe | ≤100 m | Högsby, Kalmar County, Sweden | the Global Biodiversity Information Facility (GBIF; https://www.gbif.org/) |
| *P. variegata* | 161 | 56.97 | 16.01 | Europe | ≤100 m | Högsby, Kalmar County, Sweden | the Global Biodiversity Information Facility (GBIF; https://www.gbif.org/) |
| *P. variegata* | 162 | 56.97 | 16.01 | Europe | ≤100 m | Högsby, Kalmar County, Sweden | the Global Biodiversity Information Facility (GBIF; https://www.gbif.org/) |
| *P. variegata* | 163 | 56.97 | 16.02 | Europe | ≤100 m | Högsby, Kalmar County, Sweden | the Global Biodiversity Information Facility (GBIF; https://www.gbif.org/) |
| *P. variegata* | 164 | 56.97 | 16.02 | Europe | ≤100 m | Nybro, Kalmar County, Sweden | the Global Biodiversity Information Facility (GBIF; https://www.gbif.org/) |
| *P. variegata* | 165 | 58.37 | 15.61 | Europe | ≤100 m | Nybro, Kalmar County, Sweden | the Global Biodiversity Information Facility (GBIF; https://www.gbif.org/) |
| *P. variegata* | 166 | 58.37 | 15.62 | Europe | ≤100 m | Linköping, Östergötland County, Sweden | the Global Biodiversity Information Facility (GBIF; https://www.gbif.org/) |
| *P. variegata* | 167 | 58.37 | 16.32 | Europe | ≤100 m | Linköping, Östergötland County, Sweden | the Global Biodiversity Information Facility (GBIF; https://www.gbif.org/) |
| *P. variegata* | 168 | 58.37 | 16.32 | Europe | ≤100 m | Valdemarsvik, Östergötland County, Sweden | the Global Biodiversity Information Facility (GBIF; https://www.gbif.org/) |
| *P. variegata* | 169 | 58.37 | 16.32 | Europe | ≤100 m | Valdemarsvik, Östergötland County, Sweden | the Global Biodiversity Information Facility (GBIF; https://www.gbif.org/) |
| *P. variegata* | 170 | 58.36 | 16.31 | Europe | ≤100 m | Valdemarsvik, Östergötland County, Sweden | the Global Biodiversity Information Facility (GBIF; https://www.gbif.org/) |
| *P. variegata* | 171 | 58.36 | 16.32 | Europe | ≤100 m | Valdemarsvik, Östergötland County, Sweden | the Global Biodiversity Information Facility (GBIF; https://www.gbif.org/) |
| *P. variegata* | 172 | 58.37 | 16.32 | Europe | ≤100 m | Valdemarsvik, Östergötland County, Sweden | the Global Biodiversity Information Facility (GBIF; https://www.gbif.org/) |
| *P. variegata* | 173 | 58.37 | 16.32 | Europe | ≤100 m | Valdemarsvik, Östergötland County, Sweden | the Global Biodiversity Information Facility (GBIF; https://www.gbif.org/) |
| *P. variegata* | 174 | 58.36 | 16.31 | Europe | ≤100 m | Valdemarsvik, Östergötland County, Sweden | the Global Biodiversity Information Facility (GBIF; https://www.gbif.org/) |
| *P. variegata* | 175 | 55.87 | 13.64 | Europe | ≤100 m | Valdemarsvik, Östergötland County, Sweden | the Global Biodiversity Information Facility (GBIF; https://www.gbif.org/) |
| *P. variegata* | 176 | 55.87 | 13.64 | Europe | ≤100 m | Hörby, Skåne County, Sweden | the Global Biodiversity Information Facility (GBIF; https://www.gbif.org/) |
| *P. variegata* | 177 | 57.78 | 14.24 | Europe | ≤100 m | Hörby, Skåne County, Sweden | the Global Biodiversity Information Facility (GBIF; https://www.gbif.org/) |
| *P. variegata* | 178 | 57.78 | 14.24 | Europe | ≤100 m | Jönköping, Jönköping County, Sweden | the Global Biodiversity Information Facility (GBIF; https://www.gbif.org/) |
| *P. variegata* | 179 | 57.78 | 14.24 | Europe | ≤100 m | Jönköping, Jönköping County, Sweden | the Global Biodiversity Information Facility (GBIF; https://www.gbif.org/) |
| *P. variegata* | 180 | 57.78 | 14.24 | Europe | ≤100 m | Jönköping, Jönköping County, Sweden | the Global Biodiversity Information Facility (GBIF; https://www.gbif.org/) |
| *P. variegata* | 181 | 57.78 | 14.24 | Europe | ≤100 m | Jönköping, Jönköping County, Sweden | the Global Biodiversity Information Facility (GBIF; https://www.gbif.org/) |
| *P. variegata* | 182 | 57.78 | 14.25 | Europe | ≤100 m | Jönköping, Jönköping County, Sweden | the Global Biodiversity Information Facility (GBIF; https://www.gbif.org/) |
| *P. variegata* | 183 | 57.78 | 14.24 | Europe | ≤100 m | Jönköping, Jönköping County, Sweden | the Global Biodiversity Information Facility (GBIF; https://www.gbif.org/) |
| *P. variegata* | 184 | 57.78 | 14.24 | Europe | ≤100 m | Jönköping, Jönköping County, Sweden | the Global Biodiversity Information Facility (GBIF; https://www.gbif.org/) |
| *P. variegata* | 185 | 56.78 | 14.59 | Europe | ≤100 m | Jönköping, Jönköping County, Sweden | the Global Biodiversity Information Facility (GBIF; https://www.gbif.org/) |
| *P. variegata* | 186 | 57.24 | 14.31 | Europe | ≤100 m | Alvesta, Kronoberg County, Sweden | the Global Biodiversity Information Facility (GBIF; https://www.gbif.org/) |
| *P. variegata* | 187 | 57.24 | 14.31 | Europe | ≤100 m | Alvesta, Kronoberg County, Sweden | the Global Biodiversity Information Facility (GBIF; https://www.gbif.org/) |
| *P. variegata* | 188 | 57.24 | 14.32 | Europe | ≤100 m | Värnamo, Jönköping County, Sweden | the Global Biodiversity Information Facility (GBIF; https://www.gbif.org/) |
| *P. variegata* | 189 | 57.24 | 14.31 | Europe | ≤100 m | Värnamo, Jönköping County, Sweden | the Global Biodiversity Information Facility (GBIF; https://www.gbif.org/) |
| *P. variegata* | 190 | 57.24 | 14.31 | Europe | ≤100 m | Värnamo, Jönköping County, Sweden | the Global Biodiversity Information Facility (GBIF; https://www.gbif.org/) |
| *P. variegata* | 191 | 57.24 | 14.31 | Europe | ≤100 m | Värnamo, Jönköping County, Sweden | the Global Biodiversity Information Facility (GBIF; https://www.gbif.org/) |
| *P. variegata* | 192 | 56.78 | 14.59 | Europe | ≤100 m | Värnamo, Jönköping County, Sweden | the Global Biodiversity Information Facility (GBIF; https://www.gbif.org/) |
| *P. variegata* | 193 | 56.78 | 14.59 | Europe | ≤100 m | Alvesta, Kronoberg County, Sweden | the Global Biodiversity Information Facility (GBIF; https://www.gbif.org/) |
| *P. variegata* | 194 | 56.78 | 14.59 | Europe | ≤100 m | Alvesta, Kronoberg County, Sweden | the Global Biodiversity Information Facility (GBIF; https://www.gbif.org/) |
| *P. variegata* | 195 | 59.60 | 17.02 | Europe | ≤100 m | Alvesta, Kronoberg County, Sweden | the Global Biodiversity Information Facility (GBIF; https://www.gbif.org/) |
| *P. variegata* | 196 | 59.60 | 17.02 | Europe | ≤100 m | Enköping, Uppsala County, Sweden | the Global Biodiversity Information Facility (GBIF; https://www.gbif.org/) |
| *P. variegata* | 197 | 57.23 | 14.32 | Europe | ≤100 m | Enköping, Uppsala County, Sweden | the Global Biodiversity Information Facility (GBIF; https://www.gbif.org/) |
| *P. variegata* | 198 | 57.03 | 14.02 | Europe | ≤100 m | Värnamo, Jönköping County, Sweden | the Global Biodiversity Information Facility (GBIF; https://www.gbif.org/) |
| *P. variegata* | 199 | 56.80 | 16.58 | Europe | ≤100 m | Ljungby, Kronoberg County, Sweden | the Global Biodiversity Information Facility (GBIF; https://www.gbif.org/) |
| *P. variegata* | 200 | 56.79 | 16.57 | Europe | ≤100 m | Borgholm, Kalmar County, Sweden | the Global Biodiversity Information Facility (GBIF; https://www.gbif.org/) |
| *P. variegata* | 201 | 57.68 | 11.94 | Europe | ≤100 m | Borgholm, Kalmar County, Sweden | the Global Biodiversity Information Facility (GBIF; https://www.gbif.org/) |
| *P. variegata* | 202 | 50.32 | 7.49 | Europe | ≤100 m | Gothenburg, Västra Götaland County, Sweden | the Global Biodiversity Information Facility (GBIF; https://www.gbif.org/) |
| *P. variegata* | 203 | 56.96 | 16.08 | Europe | ≤100 m | Mayen-Koblenz, Rhineland-Palatinate, Germany | the Global Biodiversity Information Facility (GBIF; https://www.gbif.org/) |
| *P. variegata* | 204 | 55.71 | 13.37 | Europe | ≤100 m | Nybro, Kalmar County, Sweden | the Global Biodiversity Information Facility (GBIF; https://www.gbif.org/) |
| *P. variegata* | 205 | 42.03 | 12.64 | Europe | ≤100 m | Gattaceca, Latium, Italy | 38. Cavallero S, Bellini I, Ascoli Bartoli T, et al. Thelaziosis in central Italy: Molecular detection of a variant haplotype in a human case and epidemiological aspects. Current Research in Parasitology and Vector-Borne Diseases. 2025;8:100316. |
| *P. variegata* | 206 | 42.62 | 11.97 | Europe | ≤100 m | Turona, Latium, Italy |  |
| *P. variegata* | 207 | 41.47 | 12.72 | Europe | ≤100 m | Foglino, Latium, Italy |  |
| *P. variegata* | 208 | 42.13 | 12.13 | Europe | ≤100 m | Manziana, Latium, Italy |  |
| *P. variegata* | 209 | 47.54 | 18.95 | Europe | ≤100 m | Ördögárok, Pest County, Hungary | 39. Erdei AL, Szelényi MO, Deutsch F, Kiss B, Molnár BP. Olfactory responses of the variegated fruit fly, *Phortica variegata*, an emerging vector of the zoonotic eyeworm *Thelazia callipaeda*, to ecologically relevant volatiles. Parasites & Vectors. 2025;18(1):204. |
| *P. variegata* | 210 | 47.65 | 18.87 | Europe | ≤100 m | Iluska Spring, Pest County, Hungary |  |
| *P. variegata* | 211 | 47.64 | 18.85 | Europe | ≤100 m | Piliscsaba, Pest County, Hungary |  |
| *P. variegata* | 212 | 42.98 | -2.63 | Europe | ≤35 km | Legutiano, Álava, Spain | 40. González MA, López-de-Felipe M, Magallanes S, et al. Distribution, identification and ecology of *Phortica* genus (Diptera: Drosophilidae) in Spain. International Journal of Veterinary Science and Medicine. 2025;13(1):1-11. |
| *P. variegata* | 213 | 42.71 | -2.49 | Europe | ≤35 km | Izki, Álava, Spain |  |
| *P. variegata* | 214 | 42.60 | -2.52 | Europe | ≤35 km | Kripan, Álava, Spain |  |
| *P. variegata* | 215 | 42.37 | -2.55 | Europe | ≤35 km | Sojuela, La Rioja, Spain |  |
| *P. variegata* | 216 | 39.65 | -5.51 | Europe | ≤35 km | Jarandilla de la Vera, Cáceres, Spain |  |
| *P. variegata* | 217 | 39.68 | -5.40 | Europe | ≤35 km | Losar de la Vera, Cáceres, Spain |  |
| *P. variegata* | 218 | 39.48 | -6.37 | Europe | ≤35 km | Cáceres, Cáceres, Spain |  |
| *P. variegata* | 219 | 42.38 | -0.72 | Europe | ≤35 km | La Peña (Water Dam), Huesca, Spain |  |
| *P. variegata* | 220 | 39.57 | 2.65 | Europe | ≤35 km | Palma, Mallorca, Spain |  |
| *P. variegata* | 221 | 40.89 | -3.56 | Europe | ≤35 km | El Berrueco, Madrid, Spain |  |
| *P. variegata* | 222 | 37.37 | -6.13 | Europe | ≤35 km | El Castillo de las Guardas, Sevilla, Spain |  |
| *P. variegata* | 223 | 37.78 | -6.37 | Europe | ≤35 km | Arroyomolinos de Léon, Huelva, Spain |  |
| *P. variegata* | 224 | 42.12 | 12.12 | Europe | ≤100 m | Rome, Latium, Italy | 9. Bernardini I, Poggi C, Porretta D, et al. Population dynamics of sympatric *Phortica* spp. and first record of stable presence of *Phortica oldenbergi* in a *Thelazia* *callipaeda*-endemic area of Italy. Parasites & Vectors. 2024;17(1):455. |
| *P. variegata* | 225 | 46.50 | 11.35 | Europe | ≤100 m | Bolzano, South Tyrol, Italy | 41. Unterköfler MS, Dengg P, Niederbacher M, et al. Occurrence of *Thelazia callipaeda* and its vector *Phortica variegata* in Austria and South Tyrol, Italy, and a global comparison by phylogenetic network analysis. Parasites & Vectors. 2023;16(1):294. |
| *P. variegata* | 226 | 48.07 | 14.06 | Europe | ≤100 m | Sattledt, Upper Austria, Austria |  |
| *P. variegata* | 227 | 48.29 | 14.25 | Europe | ≤100 m | Linz, Upper Austria, Austria |  |
| *P. variegata* | 228 | 48.34 | 14.27 | Europe | ≤100 m | Linz, Upper Austria, Austria |  |
| *P. variegata* | 229 | 47.87 | 16.56 | Europe | ≤100 m | Eisenstadt, Burgenland, Austria |  |
| *P. variegata* | 230 | 48.11 | 16.33 | Europe | ≤100 m | Wien, Vienna, Austria |  |
| *P. variegata* | 231 | 48.36 | 16.79 | Europe | ≤100 m | Gänserndorf, Lower Austria, Austria |  |
| *P. variegata* | 232 | 48.64 | 15.92 | Europe | ≤100 m | Pernersdorf, Lower Austria, Austria |  |
| *P. variegata* | 233 | 46.81 | 15.22 | Europe | ≤100 m | Schwanberg, Styria, Austria |  |
| *P. variegata* | 234 | 42.12 | 12.12 | Europe | ≤100 m | Lazio Region, Latium, Italy | 42. Bezerra-Santos MA, Bernardini I, Lia RP, et al. *Phortica oldenbergi* (Diptera: Drosophilidae): A new potential vector of the zoonotic *Thelazia callipaeda* eyeworm. Acta Tropica. 2022;233:106565. |
| *P. variegata* | 235 | 42.66 | -2.51 | Europe | ≤100 m | Álava, Basque Country, Spain | 43. González MA, Bravo-Barriga D, Alarcón-Elbal PM, et al. Development of Novel Management Tools for *Phortica variegata* (Diptera: Drosophilidae), Vector of the Oriental Eyeworm, *Thelazia callipaeda* (Spirurida: Thelaziidae), in Europe. Journal of Medical Entomology. 2022;59(1):328-336. |
| *P. variegata* | 236 | 50.84 | -1.51 | Europe | ≤100 m | Hampshire District, Hampshire, United Kingdom | 4. Pombi M, Marino V, Jaenike J, Graham-Brown J, Bernardini I, Lia RP, et al. Temperature is a common climatic descriptor of lachryphagous activity period in *Phortica variegata* (Diptera: Drosophilidae) from multiple geographical locations. Parasites & vectors. 2020;13(1):89. |
| *P. variegata* | 237 | 40.60 | -4.12 | Europe | ≤100 m | Madrid, Community of Madrid, Spain |  |
| *P. variegata* | 238 | 40.54 | 16.16 | Europe | ≤100 m | Basilicata Region, Basilicata, Italy |  |
